# Application of behavioural theories, models, and frameworks in pharmacy practice research based on published evidence: a scoping review

**DOI:** 10.1007/s11096-023-01674-x

**Published:** 2024-01-04

**Authors:** Zachariah Nazar, Lina Mohammad Naseralallah, Derek Stewart, Vibhu Paudyal, Laila Shafei, Anita Weidmann

**Affiliations:** 1 College of Pharmacy, QU Health, Qatar University, Doha, Qatar; 2 Pharmacy Department, Hamad Medical Corporation, Doha, Qatar; 3 School of Pharmacy, College of Medical and Dental Sciences, University of Birmingham, Birmingham, UK; 4 School of Pharmacy, University of Birmingham, Edgbaston, Birmingham, UK; 5 Department of Clinical Pharmacy, Innsbruck University, Innsbruck, Austria

**Keywords:** Behavioural theory, Pharmacy research, Pharmacy, Scoping review, Theoretical framework

## Abstract

**Background:**

Pharmacy practice research often focuses on the design, implementation and evaluation of pharmacy services and interventions. The use of behavioural theory in intervention research allows understanding of interventions’ mechanisms of action and are more likely to result in effective and sustained interventions.

**Aim:**

To collate, summarise and categorise the reported behavioural frameworks, models and theories used in pharmacy practice research.

**Method:**

PubMed, Cochrane Central Register of Controlled Trials (CENTRAL), Web of Science and EBSCO (CINAHL PLUS, British Education index, ERIC) were systematically searched to capture all pharmacy practice articles that had reported the use of behavioural frameworks, theories, or models since inception of the database. Results were filtered to include articles published in English in pharmacy practice journals. Full-text screening and data extraction were independently performed by two reviewers. A narrative synthesis of the data was adopted. Studies were reviewed for alignment to the UK Medical Research Council (MRC) framework to identify in which phase(s) of the research that the theory/model/framework had been employed.

**Results:**

Fifty articles met the inclusion criteria; a trend indicating an increasing frequency of behavioural theory/frameworks/models within pharmacy practice research was identified; the most frequently reported were Theory of Planned Behaviour and Theoretical Domains Framework. Few studies provided explicit and comprehensive justification for adopting a specific theory/model/framework and description of how it underpinned the research was lacking. The majority were investigations exploring determinants of behaviours, or facilitators and barriers to implementing or delivering a wide range of pharmacy services and initiatives within a variety of clinical settings (aligned to Phase 1 UK MRC framework).

**Conclusion:**

This review serves as a useful resource for future researchers to inform their investigations. Greater emphasis to adopt a systematic approach in the reporting of the use of behavioural theories/models/frameworks will benefit pharmacy practice research and will support researchers in utilizing behavioural theories/models/framework in aspects of pharmacy practice research beyond intervention development.

**Supplementary Information:**

The online version contains supplementary material available at 10.1007/s11096-023-01674-x.

## Impact statements

- There is trend indicating the increased adoption of behavioural theories/models/frameworks to underpin pharmacy practice research. However, identified articles are limited to predominantly investigations of intervention development. Therefore, we recommend that future research utilize behavioural theories/models/frameworks in phase 2–4 of the UK MRC framework.
- Pharmacy practice research will benefit from adopting a systematic approach in the reporting of the use of behavioural theories/models/frameworks.
- Inconsistent reporting of using theories/models/frameworks in pharmacy practice research has been noted among included studies, thus we suggest establishing a specific reporting checklist which could enhance the comprehensiveness of reporting and subsequently enable practitioners, policymakers, and other stakeholders to develop theory-informed interventions to promote patient safety and enhance the pharmacy practice.

## Introduction

Pharmacy practice is described as a “scientific discipline that studies the different aspects of the practice of pharmacy, and its impact on health care systems, medicine use, and patient care” [1]. It focuses on improving health outcomes of individuals and populations as well as improving access, safety, and breadth of available services [2]. Pharmacy practice research therefore embraces both clinical pharmacy and social pharmacy elements [3]. While the terms ‘clinical pharmacy’ and ‘pharmaceutical care’ have been instrumental in initiating a shift towards more person-centered approach, its distinct research scope has expanded globally to encompass clinical, behavioural, economic, and humanistic implications of the practice of pharmacy [1, 4].

A discussion paper by Nørgaard et al. in 2000 argued the need for theory-based pharmacy practice research [5]. Pharmacy practice research often focuses on the design, implementation and evaluation of pharmacy services and interventions aimed at optimising patient safety [6]. These pharmacy services all contain an element of behavioural change for the pharmacist, the patient or the wider public, to produce the desired target outcome [7]. To assist researchers, the UK MRC Framework, first published in 2000, provides a structured approach to develop, evaluate, and implement such complex interventions using a range of qualitative, quantitative and mixed-method research approaches to help researchers make appropriate methodological and practical choices [8]. The UK MRC framework recognizes four phases of complex intervention research: 1. Development or identification of an intervention; 2. Assessment of feasibility of the intervention and evaluation design, 3. Evaluation of the intervention, 4. Impactful implementation [8]. They advocate underpinning theory at each phase.

Underpinning studies with behavioural theories/models/frameworks, has the potential to assist researchers to better understand the behaviour change process and guide the refinement of the intervention [9].

Many behavioural change theories/models/frameworks exist in the application of healthcare research. As a result, identifying the most suitable behavioural theory/model/framework to adequately address the desired research question is difficult and requires the appropriate expertise and a comprehensive understanding of available theories, models and frameworks. This starts with a correct understanding of the terminologies used.

### Theories, models, and frameworks explained

Although there are many explanations of theories, models, and frameworks, there are many similarities and overlapping concepts. One common definition of ‘theory’ is “…an account of the world, which goes beyond what we can see and measure. It embraces a set of inter-related definitions and relationships that organises our concepts and understanding of the empirical world in a systematic way” [10]. A good theory provides a clear explanation of how and why specific relationships lead to specific events [11].

A model is often a simplified representation of a complex system, designed to focus on a specific question [12]. Models can be described as theories with a more narrowly defined scope of explanation; a model is descriptive, whereas a theory is explanatory as well as descriptive [13]. Models need not always be completely accurate representations of reality to be of value [14]. According to Creswell, a complex research theory may be presented as a simplified model so “that the reader can visualize the interconnections of variables” [15]. A conceptual framework on the other hand provides a set of “big” or “grand” concepts or theories [16]; frameworks do not provide explanations; they categorise empirical phenomena [13].

Supplementary Material 1 aims to provide a brief overview of some of the behavioural theories/models/frameworks commonly used in healthcare research. Bandura’s Social Cognitive Theory proposes that people are driven by external factors rather than inner forces [17]; the Theory of Planned Behaviour is dependent on one’s intention to perform the behavior [18], while the Transtheoretical Model proposes change as a process of six stages [19]. The COM-B model allows the mapping of the capability, opportunity and motivation of any person to determine the likelihood of a behaviour to occur [20]. The Theoretical Domains Framework (TDF), an *“*integrative framework developed from a synthesis of psychological theories as a vehicle to help apply theoretical approach to intervention aimed at behavioural change”, is useful to better understand implementation problems of health initiatives which are often heterogeneous and complex [21].

The recently articulated Granada statements published in a number of clinical and social pharmacy practice journals aspire to improve the quality of publications and advance the paradigms of related pharmacy practice research [3]. It is therefore timely to review the use of behavioural theories/models/frameworks in pharmacy practice research to date to inform future studies.

### Aim

The aim of this scoping review was to collate, summarise and categorise the reported behavioural theories/models/frameworks used in pharmacy practice research.

## Method

### Protocol and registration

This scoping review was conducted and reported in accordance with the Preferred Reporting Items for Systematic reviews and Meta-analysis extension for scoping review (PRISMA-ScR) guidelines [22]. The protocol was registered in the Open Science Framework database (Registration number: qfw6d).

### Eligibility criteria

The review included studies published in pharmacy practice journals. A list of the 33 peer-reviewed pharmacy practice journals indexed in PubMed, was compiled based on Mendes et al.’s study, which classified 285 pharmacy journals into six clusters including ‘Pharmacy Practice’ (67 journals, 33 indexed in PubMed) [23]. (Supplementary Material 2).

Databases were searched since inception to capture all pharmacy practice articles that had reported the use of any behavioural theories/models/frameworks. If it was not immediately clear whether the theory/model/framework was eligible for inclusion, consensus was sought between two research team members (ZN and LN) with reference made to the research that described the theory/model/framework development, if necessary. Consultation with the wider research group was made if consensus could not be reached.

Only studies published in English were included. All primary research study designs and reviews were considered. Letters, commentaries, perspectives, and editorials were excluded, as were studies that developed and/or validated theories.

### Information sources and search strategy

The following electronic databases were independently searched by two authors (ZN, LN) on 30 May 2022; PubMed, Cochrane Central Register of Controlled Trials (CENTRAL), Web of Science and EBSCO (CINAHL PLUS, British Education index, ERIC). The following search string was used for PubMed and adapted for the other databases: (pharmacy(MeSH) [Title/Abstract]) AND ((theor*[Title/Abstract]) OR (framework [Title/Abstract])). Search strategies are provided in Supplementary Material 3.

Articles were exported to Rayyan QCRI® [24] and duplicates removed. Filters were applied to include articles published in the aforementioned 33 Pharmacy Practice Journals. Title/abstract screening and full-text screening were independently performed by two reviewers (ZN, LN). In cases of disagreements a third reviewer was consulted. Reference lists of included studies were manually checked.

### Data charting process and data items

The authors designed a data extraction tool based on the inclusion criteria and focused on key information required to comprehensively answer the research question and piloted it with 3 included articles. The following data were extracted: country, year of publication, study type and design, objective of study, outcomes measured, and the theory/model/framework reported in the study. Further details regarding how the theories/models/frameworks were used in study design including the research phase, context, and purpose of its use, were also extracted. Six reviewers were involved in the data extraction process and data extraction of each article was performed independently by two reviewers. In cases of disagreements a third reviewer was consulted.

### Synthesis of results

Data were summarized quantitatively and qualitatively in relation to the research aim. Descriptive statistics were used to describe the number of studies by year published, country, and research design. Summary statistics were used to report the frequency of use, rationale for use, and how each theory/model/framework was used in the reported studies. A narrative approach was adopted to synthesise the findings. Narrative synthesis has been defined as “an approach to the systematic review and synthesis of findings from multiple studies that relies primarily on the use of words and texts to summarize and explain the findings of the synthesis” [25].

Further, studies reporting a complex intervention as defined by the UK MRC, as those with several interacting components, or if they are dependent on the behaviour of those delivering and receiving the intervention [8], were reviewed to identify in which phase(s) of complex intervention research the theory/model/framework had been employed.

## Results

### Search results

**Fig. 1:**
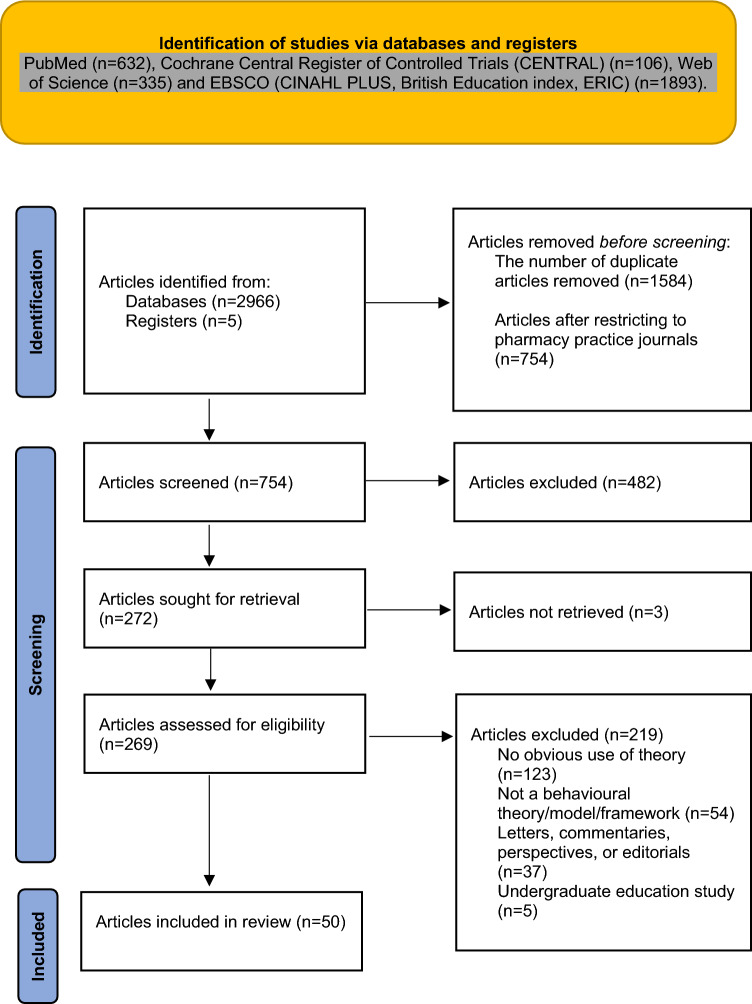
PRISMA diagram of study selection and inclusion

Fifty articles met the inclusion criteria (Fig. 1 presents the PRISMA Flow Diagram). A summary of the characteristics of included studies is presented in Table 1 and Supplementary Material 4 provides full details of the included studies.

**Table 1:** Summary of the characteristics of included studies (n = 50)

Geographical dispersion of the studies. n = number of studies, (% of the included studies)			
North America	21 (42%)	Oceania/Australia	8 (16%)
Europe	9 (18%)	Africa	2 (4%)
Asia	8 (16%)	Not applicable/Not stated	2 (4%)
Setting in which the studies were conducted. n = number of studies, (% of the included studies)			
Community pharmacies	30 (60%)	Primary care	3 (6%)
Multiple settings	12 (24%)	Not stated	2 (4%)
Hospital (inpatient and outpatient)	3 (6%)		
Study population. n = number of studies, (% of the included studies)			
Pharmacy workforce	31 (62%)		
Patients	12 (24%)	Physicians	1 (2%)
Multiple stakeholders	5 (10%)	Not applicable	1 (2%)
Methods adopted in the included studies. n = number of studies, (% of the included studies)			
Quantitative (survey)	18 (36%)	Mixed methods	9 (18%)
Qualitative (interviews)	15 (30%)	Systematic review	1 (2%)
Qualitative (focus groups)	4 (8%)	Others (mapping, exploratory descriptive)	2 (4%)
Qualitative (focus groups and interviews)	1 (2%)		
Theory/model/framework adopted. n = number of studies, (% of the included studies)			
TPB	18 (36%)	Miscellaneous	7 (14%)
TDF	11 (22%)	COM-B	3 (6%)
Multiple theories	9 (18%)	HBM	2 (4%)

TBP: Theory of planned behaviour; TDF: theoretical domain framework; COM-B: capability, opportunity, and motivation behavioural model; HBM: health belief model

### Study characteristics

**Fig. 2:**
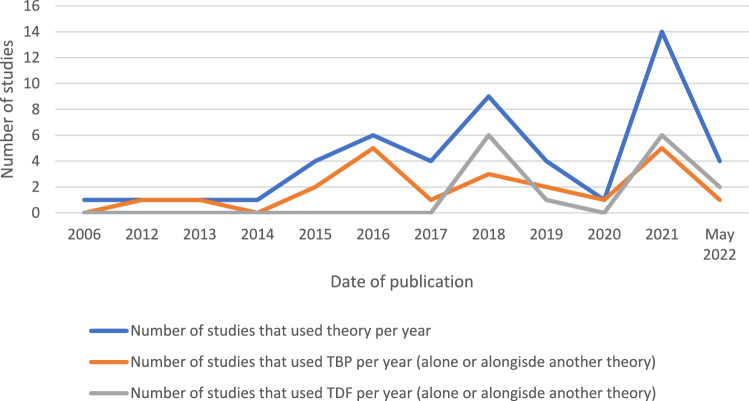
The number of pharmacy practice studies adopting behavioural theory/model/framework since 2006

Included studies were published between 2006 and 2022, with a marked rise after 2014 (Fig. 2). Most studies were conducted in North America (n = 21) and in community pharmacies (n = 30). Study subjects included pharmacy workforce (n = 31), patients (n = 12), multiple stakeholders (n = 5), and physicians (n = 1) (Table 1).

Twenty studies were qualitative (primarily individual interviews), eighteen cross-sectional surveys and nine mixed-methods. Only one systematic review related to pharmacy practice reported utilizing a theory for data synthesis [26]. Tables 2, 3, 4 present details describing the aim of the included studies; the majority of the studies were investigations of pharmacy complex interventions, as defined by the UK MRC framework. These included investigations to explore pharmacists’ involvement in various initiatives such as medicines optimization services [27], immunization clinics [28], pharmacist prescribing [29], falls prevention [30], medicines management services [31–35], and pharmacogenomics testing [36, 37].

**Table 2:** Summary of how the theory of planned behaviour (TBP) was used in the included studies (n = 18)

Authors	Study design	Study population	Purpose of using theory/model/framework	Aspect of the research using theory/model/framework
Salgado et al. [56]	Qualitative, semi-structured interviews	Renal-specialized hospital pharmacists	To investigate intentions to implement pharmacy services in outpatient dialysis centers	Interview guide, data analysis & interpretation
DeMik et al. [70]	Quantitative, cross-sectional survey	Physicians & pharmacists	To investigate behavioural determinants to implement new pharmacy clinical service	Questionnaire design, data analysis & interpretation
Amin et al. [44]	Quantitative, cross-sectional survey	Community pharmacists	To investigate medication regimen adjustment behavioural for patients during Ramadan	Integrated into research question, study design, data collection, analysis & interpretation
Kennelty et al. [43]	Qualitative, semi-structured interviews	Community pharmacists	Explore the barriers & facilitators faced when reconciling medications	Interview guide, data analysis & interpretation
Amin et al. [62]	Quantitative; cross-sectional survey	Community pharmacists	To predict counseling on oral contraceptives (OCs)	Questionnaire design, data analysis & interpretation
Lee et al. [63]	Quantitative; cross-sectional survey	Asthma patients	To examine the influences, motivation, and self-efficacy to collect asthma controller medications from the pharmacy	Survey design, data analysis & interpretation
Puspitasari et al. [67]	Quantitative; cross-sectional survey	Community pharmacists	To investigate attitudes to delivering cardiovascular disease (CVD) support; and the environmental factors that influence the provision of CVD care	Questionnaire design, data analysis & interpretation
Tan et al. [45]	Mixed methods; semi-structured interviews, survey	Patients	To explore the key determinants and mediators of successful implementation of new public pharmaceutical services	Data analysis & interpretation
Amin et al. [57]	Qualitative; semi-structures interviews	Community pharmacy staff	To examine factors associated with the unwarranted dispensing of subtherapeutic doses of antibiotics	Interview guide, data analysis & interpretation
Adeoye et al. [39]	Quantitative; cross-sectional survey	Community pharmacy staff	To investigate association between attitudes and planned behaviourals toward participating in medication therapy management services	Questionnaire design
George et al. [59]	Qualitative; focus group	Community pharmacists	To explore factors associated beliefs to improve Star Ratings	Interview guide, data analysis & interpretation
Humphries et al. [58]	Qualitative; focus groups and individual interviews	Hospital & community pharmacists	To identify attitudinal, normative, and control beliefs regarding adjuvant endocrine therapy adherence	Interview guide
Fleming et al. [46]	Qualitative; focus groups	Community pharmacists	To investigate beliefs regarding willingness to engage patients with suspected controlled substance misuse	Interview guide
Hasan et al. [49]	Quantitative; survey	Community pharmacy patients	To understand factors influencing intention to seek pharmacist-provided medication therapy management services	Questionnaire design
Falope et al. [48]	Qualitative; interviews	Pharmacists	To explore the perceptions and knowledge of administering inactivated influenza vaccines (IIV) to pregnant women	Interview guide
Gülpinar et al. [47]	Qualitative; semi-structures interviews	Community pharmacy	To explore conscientious objection to provide pharmacy services that are contrary to personal beliefs	Interview guide, data analysis & interpretation
Nichols et al. [38]	Quantitative; cross-sectional survey	Community pharmacy preceptors	Investigate experiences, clinical and legislative knowledge, attitudes, and behaviourals regarding cannabidiol	Questionnaire design
Wash et al. [53]	Qualitative; focus groups	Pharmacists (& 1 pharmacy student)	To identify factors that influence intention to prescribe	Interview guide, data analysis & interpretation

**Table 3:** Summary of how the theoretical domains framework (TDF)) was used in the included studies (n = 11)

Authors	Study design	Study population	Purpose of using theory/model/framework	Aspect of the research using theory/ model/framework
Cardwell et al. [32]	Qualitative; semi-structures interviews	Community pharmacists	To identify facilitators and barriers towards the utilization of a screening tool as a guide to conducting structured medicines use review (MURs)	Interview guide
Isenor [29]et al.	Quantitative; cross-sectional survey	Licensed pharmacists that are members of the Pharmacy Association of Nova Scotia	To identify barriers and facilitators to pharmacist prescribing	Questionnaire development
Rushworth et al. [50]	Quantitative; survey	Patients	To identify issues of access to general practitioners, community pharmacies and prescribed medicines in older people resident in the Scottish Highlands	Questionnaire development
Seubert et al. [55]	Mixed methods; systematic review, focus groups, intervention development, intervention feasibility study	Pharmacy staff and consumers	To identify barriers and facilitators for information exchange during OTC consultations in community pharmacies	Data analysis
Seubert et al. [54]	Mixed methods study; audio-recording, OTC consultations, consumer questionnaires and interviews, and pharmacy personnel interviews	Pharmacy staff and consumers	To explore intervention functions and the resulting behavioural change techniques that would most suitably address these barriers were identified	Data analysis and interpretation
Paudyal et al. [40]	Quantitative; survey	Community pharmacists	To determine community pharmacists’ training, experiences and behavioural determinants in counselling and management of homeless population	Questionnaire development
Hussein et al. [65]	Mixed methods; survey, semi-structured interviews	Pharmacists	To identify barrier and facilitators influencing the adoption of full scope services among pharmacy professionals	Questionnaire and interview design, data, triangulation of findings
Mohammed et al. [66]	Qualitative; interviews	Pharmacists	To explore pharmacist’s perceptions, current opportunities and key challenges towards the uptake of non-traditional roles	Data analysis
Patton et al. [31]	Mixed methods; semi-structured interviews, cross-sectional survey	Community pharmacists	To identify barriers and facilitators influencing community pharmacists’ provision of medication adherence support (MAS) to older patients prescribed multiple medications	Interview guide, questionnaire development, data analysis and interpretation
Alenezi et al. [27]	Qualitative; semi-structures interviews	Community pharmacists	To explore pharmacists' roles, barriers and determinants related to pharmacists’ involvement in optimizing prescribed opioids for patients with chronic pain	Interview guide
Lindner et al. [28]	Quantitative; cross-sectional survey	Community pharmacists	To identify relevant requirements and barriers to implementation of an immunization service and desired training specifications	Questionnaire development

**Table 4:** Summary of how other theories were used in the included studies

Authors	Theory/model/framework used	Study design	Study population	Purpose of using theory/model/framework	Aspect of the research using theory/ model/framework
Studies that used the COM-B model					
Hattingh et al. [51]	COM-B	Qualitative; semi-structures interviews	Community pharmacists	To explore the factors that contributed to the successful implementation and ongoing provision of enhanced and extended services in Western Australian community pharmacies	Data analysis
Bertilsson et al. [33]	COM-B	Qualitative; semi-structures interviews	Community pharmacists	To identify factors affecting recruitment of patients in community pharmacies participating in a multicenter trial of a pharmacy asthma service in Australia	Data analysis
Gemmeke et al. [30]	COM-B	Mixed methods; survey, interviews	Community pharmacists	To identify barriers and facilitators in offering fall prevention services including deprescribing of fall risk-increasing drugs (FRIDs)	Data analysis and interpretation of the qualitative part
Studies that used the HBM					
Pinto et al. [68]	HBM	Quantitative, cross-sectional survey	Patients	To determine factors affecting patient retention in pharmaceutical care services	Questionnaire development
Alatawi et al. [34]	HBM	Quantitative; cross-sectional survey	Patients	(1) assess self-report of medication-taking in a Saudi T2D convenience sample, (2) investigate self-reported HBM constructs for T2D, its complications, and medication-taking in this sample, and (3) test the ability for self-reported health beliefs to predict specific medication-taking behaviourals among the sample	Questionnaire development, data interpretation and discussion
Studies that used other theories/models/frameworks					
Odukoya et al. [71]	Three-step error recovery model	Mixed methods; observations, interviews, focus groups	Community pharmacists	To describe the process used by community pharmacy staff to detect, explain, and correct e-prescription errors	Interview guide and data analysis
Desai et al. [64]	Andersen Behavioural Model	Quantitative; cross-sectional survey	Consumers	To identify factors associated with accessing medications/vitamins online and to identify factors associated with discussions of online information	Data analysis
Ziaei et al. [60]	Model of Communicative Proficiency (MCP)	Qualitative, focus groups	Pharmacists (community and hospital)	To investigate Internationally Trained Pharmacists (ITPs’) perceptions of their communication proficiency and the resultant impact on patient safety	To understand the findings
Chevalier B et al. [61]	Communication Accommodation Theory (CAT)	Qualitative; semi-structures interviews	Pharmacists and patients (inpatient and outpatient)	To explore hospital pharmacists’ and patients’ views about what constitutes effective communication exchanges between pharmacists and patients	Data analysis
Murad et al. [52]	Face-work theory	Exploratory descriptive design	Community pharmacists and customers	To determine face needs, threats and the strategic communication strategies used to address these within community pharmacist-patient interactions	Data analysis
Waddell et al. [69]	Alimo-Metcalfe and Alban-Metcalfe model of transformational leadership	Mapping	Not applicable	To map the leadership and management domain of the Australian Advanced Pharmacy Practice Framework (APPF) against the model of transformational leadership and make comment on the potential utility of the APPF to develop advanced practitioners in the area of leadership and management	To map the Australian framework
Qudah et al. [26]	Street’s Linguistic Model of Patient Participation in Care (LM)	Systematic review	Community pharmacists	To identify barriers and facilitators of patients' engagement in pharmacy consultations	Data synthesis (mapping)
Studies that used multiple theories/models/frameworks					
Luder et al. [73]	HBM, TPB, and Theory of Reasoned Action (TRA)	Quantitative; cross-sectional survey	Community pharmacists	To describe the characteristics, health beliefs, and cues to action of newly enrolled participants	Questionnaire development
Fingleton et al. [72]	TDF and COM-B model	Mixed methods; survey, semi-structured interviews	Doctors	To establish how non-prescription medicine (NPM) dependence is treated by doctors in specialist substance misuse treatment services and to identify perceived barriers to providing treatment	Questionnaire development and interview topic guide
Jonkman et al. [41]	HBM, TPB, and the Explanatory Models of Illness (EMI)	Qualitative; semi-structures interviews	Public hospitals, private hospitals, and community pharmacies	To identify patient-reported barriers and facilitators to managing chronic non-communicable diseases (NCDs) and characterize medication and health related needs with chronic NCDs	Interview guide design and data analysis
Abdu-Aguye et al. [75]	TDF and COM-B model	Qualitative; semi-structures interviews	Outpatient pharmacies located within hospitals	To understand barriers/facilitators to optimal medication counselling by conducting a behavioral analysis using the COM-B model, TDF, and BCW	Interview guide design and data analysis
Bright et al. [37]	Rubin and Rubin framework and TPB	Qualitative; semi-structures interviews	Community pharmacists	To identify patient perceptions related to pharmacogenomic testing in the community pharmacy setting	Interview development
Faisal et al. [35]	The Technology Acceptance Model (TAM), TPB, and COM-B model	Mixed methods; semi-structured interview, survey	Community pharmacists	To explore factors affecting implementation of a real-time adherence-monitoring, multidose-dispensing system in community pharmacies	Interview development; analysis (mapping) of themes
Luke et al. [36]	TDF and COM-B model	Qualitative; semi-structures interviews	Pharmacists who completed the PRIME training program irrespective of setting	To elucidate the factors influencing the integration of pharmacogenomics testing by pharmacists in their practices	Data analysis
Viegas et al. [74]	TDF and COM-B model	Quantitative; cross-sectional survey	Community pharmacists	To characterize the major facilitators and barriers faced by pharmacists in their daily practice	Questionnaire development
Okuyan et al. [42]	Transtheoretical model of behavior change and HBM	Quantitative; cross-sectional survey	Community pharmacists	To determine the intention to receive COVID-19 vaccine and to identify the factors related to it based on the HBM framework among Turkish pharmacists	Questionnaire development

COM-B: capability, opportunity, and motivation behavioural; HBM: health belief model; TPB: theory of planned behaviour; BCW: behavioural change wheel

### Theories, models, frameworks used

Tables 2, 3, 4 present the data pertaining to how theories/models/frameworks were used in the included studies. The majority (n = 39) of studies used a single theory/model/framework, most commonly the Theory of Planned Behaviour (TPB) (n = 18), followed by the Theoretical Domains Framework (TDF) (n = 11). In studies using a combination of multiple theories/models/frameworks; the most frequent combination was TDF with the Capability, Opportunity, and Motivation Behaviour (COM-B) model.

### Justification for theories/models/frameworks selected

Multiple justifications were reported for the use of theories/models/frameworks however, reporting was inconsistent, for example multiple studies simply mentioned that the theory/model/framework guided the development of the data collection tool [32, 37–42]. Beyond this, 14 studies provided a description of the theory/model/framework constructs and/or assumptions but without connecting it to the research question [28, 43–55]. Nine studies provided the justification that the theory/model/framework had been used previously in similar research or within the same field [27, 30, 33, 56–61]. Only seven studies connected the theory/model/framework with the research question of the study [26, 29, 31, 34, 62–64]. Other reasons provided included the potential/predicted benefits the theory/model/framework might have on the findings (n = 3) [65–67]; recommendation from leaders in the field (n = 2) [68, 69]; and the absence of theory-informed studies in the existing body of literature (n = 2) [70, 71].

Studies that combined multiple theories/models/frameworks cited their potential synergies as the chief driver for their combined use (n = 3) [35, 36, 72] however six studies did not provide a rationale for the combination [37, 41, 42, 73–75].

### How theories/models/frameworks were used

The use of most theories/models/frameworks (n = 31) aligned to Phase 1 of the MRC framework; to explore determinants of behaviours, or facilitators and barriers to implementing or delivering new pharmacy services. Eighteen of these studies proceeded to identify theoretical domains that should be targeted in future interventions aimed at behavioural change. Three studies [35, 54, 55] aligned to Phase 2 of the MRC framework where the theory/model/framework was used in assessing intervention feasibility. However, there was a lack of detail to determine how the theory underpinned this assessment. Studies to evaluate an intervention and to assess the impact of an intervention (Phase 3 and 4 of the MRC framework) were not identified in this review. Most theories/models/frameworks were used to inform the item development of the data collection tool (n = 24) followed by guiding data analysis (n = 17). A large number of studies used theories (n = 20) in multiple aspects of the research, in most cases to inform the data collection tool then in the subsequent data analysis and interpretation. An example includes a study that used TPB in constructing interview questions to examine the barriers and facilitators reported by community pharmacists when reconciling medications for patients recently discharged from hospital. The subsequent analysis generated themes organized based on the TPB constucts [43].

The following sections provide descriptions specific to how each of the most common theories/models/frameworks were utilized in the included studies.

### Theory of planned behaviour (TPB)

TPB was used in 21 studies (Table 2 provides a summary of how TPB was used in 18 of these studies, in the other three studies TPB was used alongside a second theory/model/framework, details of studies which combined multiple theories/models/frameworks can be found in Table 4). Of the 18 studies, 15 were conducted with pharmacy professionals, in the most part to investigate behavioural influences to either implement or deliver pharmacy service initiatives (examples include vaccination services, medication therapy management services, cardiovascular support) or specific aspects of pharmaceutical care (examples included medication counselling, clinical decision making, ethical dilemmas). Three studies were conducted with patients, their focus was to understand patient behaviours in seeking pharmacy services. Although not explicitly mentioned in the majority of reports, the intervention studies aligned to Phase 1 of the UK MRC framework. TPB was used to guide the design of the data collection tool in majority of studies and less frequently to guide the analysis and interpretation of the collected data.

### Theoretical domains framework (TDF)

TDF was used in 15 studies (Table 3 provides a summary of how TDF was used in 11 of these studies, in the other 4 studies TDF was used alongside a second theory/model/framework, details of these studies are presented in Table 4). Eight of the 11 studies were conducted with pharmacy professionals, in the most part to identify facilitators and barriers to either implement or deliver pharmacy service initiatives (examples include independent prescribing and immunization clinics) or specific aspects of pharmaceutical care (for example medication counselling). Thirteen studies aligned to Phase 1 of the UK MRC framework, the two other studies [54, 55] were research articles presenting data from the same project which aimed to assess the feasibility of delivering extended pharmaceutical care in community pharmacies in Australia.

### Capability, opportunity, and motivation behaviour (COM-B) model

COM-B was used in 8 studies (Table 4 provides a summary of how COM-B was used in 3 of these studies, in the other 5 studies COM-B was used alongside another theory/model/framework, details of which are also presented in Table 4). All studies that used COM-B were conducted with community pharmacists to explore behavioural determinants to implement pharmacy services initiatives (these included a fall prevention service, extended pharmaceutical care services, and an asthma management service) and aligned to Phase 1 of the MRC framework. In all studies COM-B was used exclusively in the data analysis.

### Health belief model (HBM)

HBM was used in 5 studies (Table 4 provides a summary of how HBM was used in 2 of these studies; in the other 3 studies HBM was used alongside another theory/model/framework, details of which are also presented in Table 4). All studies that used HBM were conducted with patients to explore behavioural determinants and predict behaviours. The studies aligned to Phase 1 of the UK MRC framework. In all studies, HBM was used for questionnaire development.

### Studies that used multiple theories/models/frameworks

Other than studies utilizing TDF, which is a comprehensive framework derived from 33 psychological theories and 128 theoretical constructs [21], there were nine studies that combined multiple theories/models/frameworks. (Table 4). All studies that combined multiple theories were conducted with pharmacy professionals except for one with physicians investigating a substance misuse treatment service [72]. The primary purpose for conducting these studies was to explore behavioural determinants to implement pharmacy-based services. However, one study that described a service to treat non-prescription medication dependence used TDF and COM-B to establish the physicians’ behaviours that should be targeted in an intervention [72]. These studies aligned to Phase 1 of the UK MRC framework.

### Other theories/models/frameworks

Thirteen other behavioural theories/models/frameworks were adopted in the included studies, seven were used alone and six were combined with one of the aforementioned theories/models/frameworks. The justification and purpose for use of these theories/models/frameworks was inconsistently described. For instance, the Model of Communicative Proficiency (MCP) was used in a study to frame the findings but there was no consideration of its integration into the study methodology [60]. Exceptions to this were (n = 3) using the Andersen Behavioural Model [64], Explanatory Models of Illness (EMI) [41], and Alimo-Metcalfe and Alban-Metcalfe Model of Transformational Leadership [26]. The use of these theories/models/frameworks were thoroughly described and were incorporated in the design, analysis, and results synthesis and interpretation. In these studies theories were used to identify the determinants of behaviour to target in future interventions.

### Reported benefits and challenges of using a theory/model/framework

Multiple studies described the benefits of using a theory/model/framework. Most frequently mentioned was the use of theory facilitating a more comprehensive understanding of the phenomenon under investigation compared to existing similar interventions; and secondly, the use of theory elucidated specific psychosocial factors influencing health-related behaviours and provided avenues for future research into targeted intervention development and relevant policy to improve practice or enhance patient safety. In contrast, the challenges authors faced in using theory/model/framework were rarely reported in the manuscript.

## Discussion

### Summary of key findings

This study identified the increasing trend to adopt the use of behavioural theories/models/frameworks within pharmacy practice research. The most utilized behavioural theories reported in pharmacy practice studies were the most established: Theory of Planned Behaviour (TPB); Theoretical Domains Framework (TDF): Capability, Opportunity, and Motivation Behaviour (COM-B) model; and the Health Belief Model (HBM). These findings are consistent with reviews conducted in other health domains [76–78]. Few studies provided explicit and comprehensive justification for adopting a specific theory/model/framework.

The majority of the included studies were investigations exploring determinants of behaviours, or facilitators and barriers to implementing or delivering a wide range of pharmacy services and initiatives within a variety of clinical settings. In reviewing the use of behavioural theories/models/frameworks against the four phases of complex intervention research proposed in the UK MRC framework, it was determined that most studies were focused on developing an intervention within a pharmacy setting (Phase 1), very few studies aligned to Phases 2–4 of the UK MRC framework.

### Strengths and limitations

This scoping review was conducted through the application of rigorous and transparent processes [22, 79] and to the best knowledge of the authors, is the first review that reports the use of behavioural theories/models/frameworks in pharmacy practice research. One limitation is that the review was restricted to articles published in the English language only, and articles published in 33 ‘Pharmacy Practice’ journals hence relevant publications in other languages, and in other pharmacy and non-pharmacy journals were not included. Also, investigating the gaps in the theories/models/frameworks that have been applied to pharmacy practice research fell outside the scope of this review, but the authors agree that this would be a worthwhile follow-up study.

### Interpretation of findings

The majority of the included studies reported on interventions within pharmacy practice. Whilst there were many studies investigating determinants of behaviours, or facilitators and barriers to implementing new services (phase 1 of the MRC framework); there were substantially fewer studies reporting on the subsequent phases of the MRC framework. There is evidence to suggest that studies of intervention feasibility, evaluation and implementation are frequently published in journals other than pharmacy practice journals. For example a 2020 systematic review of interventions using health behaviour theories to improve medication adherence among patients with hypertension, included 11 studies, none of which were published in pharmacy practice journals [80]. The same finding was found from a 2022 systematic review to determine the utilization of the transtheoretical model of change, to predict or improve medication adherence in patients with chronic conditions [81]. Although, it is possible that publishing in non-pharmacy practice journals may enhance the visibility of the research, it means that pharmacy practice journals do not benefit from the potential impact of this research. Furthermore, the Granada statements encourage researchers to prioritise pharmacy practice and social pharmacy journals for some of their “best” work with the aim to strengthen the discipline of pharmacy practice research [3].

The use of a behavioural theory/model/framework to underpin data collection tools and data analysis in the included studies, was reported to elicit greater insight of behavioural determinants compared to existing literature that had not adopted this approach. This broader assessment was often claimed to have helped in identifying potentially unknown behavioural influences which can be targeted in the design of interventions. However, beyond describing how theory was used to inform questionnaire-design, studies lacked explicit detail of how the theory was used to underpin data analysis and interpret study findings. It is plausible, as suggested elsewhere in the literature, that word limits imposed by journals may restrict the provision of information on theoretical underpinning [82]. However, the lack of detail included meant that it was often difficult to determine what theoretical components and strategies were associated with the success or challenges of the intervention. Thus, we recommend the inclusion of further detail relating to theoretical underpinnings and expected causal mechanisms of behavioural change prospectively, and evaluation of these mechanisms to better understand what strategies are effective. This would facilitate evidence synthesis, prevent research duplication and enhance transferability of study findings [83, 84].

Moreover, since it is well-established that the use of theory in intervention research allows understanding of interventions’ mechanisms of action and are more likely to result in effective and sustained interventions [83, 85], greater consistency in describing the rationale for theory selection is warranted, with recognition that different theories are more applicable to different study settings. Selecting the most appropriate theory from amongst the wide range of options, is likely to be perplexing for researchers [86, 87], thus, guidelines to direct researchers in this regard may also serve as a useful resource. The use of checklists such as the Template for Intervention Description and Replication (TIDieR), which includes an item to describe any theory used in studies when describing an intervention, has been developed to improve the completeness of reporting, and ultimately the replicability, of interventions [88]. Also authors may consider the use of a tool recently developed by Michie and Prestwich, the Theory Coding Scheme(TCS), which assesses the degree to which an intervention uses theory to guide intervention design, implementation and evaluation [89]. This tool includes 10 specific coding criteria, which range from noting whether a theory was mentioned in the introduction of a journal article to whether the findings of the study were discussed in a theoretical context. This tool may serve as a useful framework for authors to improve the use of theory and act as a blueprint for the design and reporting of intervention studies.

### Further work

With the growing use of behavioural theory in pharmacy practice research, studies to ascertain whether theories/models/frameworks are being used correctly are warranted. For example, constructs may be misinterpreted or poorly measured which may result in inappropriate analysis. Such studies will help to provide further guidance for researchers.

Furthermore, this review has highlighted the inconsistent reporting of using theories/models/frameworks in pharmacy practice research, thus suggesting potential advantage to establish a specific reporting checklist.

Finally, this review did not elicit the challenges researchers face in using behavioural theory to underpin their studies, further investigations are necessary to explore these issues.

## Conclusion

Behavioural theory/models/frameworks are increasingly being adopted to underpin pharmacy practice research across a variety of research designs and frequently in studies of initial investigations of complex interventions within various settings. The findings from this review indicate the need for more thorough reporting in regards to the rationale for the selection of a specific behavioural theory/model/framework; details of its application in underpinning the research; and the challenges and limitations encountered. Clearer reporting will aid in determining how best to use behavioural theory/models/frameworks in pharmacy practice research. Furthermore, studies adopting behavioural theories/models/frameworks in the latter stages of interventional research (feasibility testing, evaluation and implementation) published in pharmacy practice journals will help to further strengthen the field.

### Supplementary Information

Below is the link to the electronic supplementary material.

Supplementary file1 (DOCX 76 KB)
