# Supplementary material for: Application of behavioural theories, models, and frameworks in pharmacy practice research based on published evidence: a scoping review
Source: Int J Clin Pharm. 2024 Jan 4;46(3):559–73. doi: 10.1007/s11096-023-01674-x (PMC11133055; doi:10.1007/s11096-023-01674-x)
Supplement: Supplementary file 1 — Supplementary file1 (DOCX 76 KB) [file 11096_2023_1674_MOESM1_ESM.docx]

**Supplementary Material 1: A brief overview of some of the behaviour change theories, models and frameworks commonly used in pharmacy practice research**

| Name | Description |
| --- | --- |
| Theory of Planned Behaviour (TPB)/ Theory of Reasoned Action (TRA) (1) | The Theory of Planned Behavior (TPB) started as the Theory of Reasoned Action in 1980 to predict an individual's intention to engage in a behavior at a specific time and place. The theory was intended to explain all behaviors over which people have the ability to exert self-control. |
| Theoretic Domains Framework  (TDF) (2) | The Theoretical Domains Framework (TDF) is an integrative framework developed from a synthesis of psychological theories as a vehicle to help apply theoretical approaches to interventions aimed at behavior change. |
| Transtheoretical Model of Behaviour change (3) | The transtheoretical model posits that health behavior change involves progress through six stages of change: precontemplation, contemplation, preparation, action, maintenance, and termination. |
| The Capability Opportunity Motivation- Behaviour model (COM-B) (4) | According to the COM-B model, for a given behaviour to occur, at a given moment, one must have the capability and opportunity to engage in the behaviour, and the strength of motivation to engage in the behaviour must be greater than for any other competing behaviour. |
| Health belief Model (5) | Suggests that a person's belief in a personal threat of an illness or disease together with a person's belief in the effectiveness of the recommended health behavior or action will predict the likelihood the person will adopt the behavior. |
| Andersen Behaviour Model (6) | The Andersen Model was originally proposed to present a theoretical framework to understand and explain how and why people use certain types of health services or general types of health services. |

1. LaMorte WW. Behavioral change models: The theory of planned behavior. Boston University School of Public Health Available online at: https://sphweb bumc bu edu/otlt/mph-modules/sb/behavioralchangetheories/BehavioralChangeTheories3 html (accessed June 02, 2022). 2019.
2. Phillips CJ, Marshall AP, Chaves NJ, Jankelowitz SK, Lin IB, Loy CT, et al. Experiences of using the Theoretical Domains Framework across diverse clinical environments: a qualitative study. Journal of multidisciplinary healthcare. 2015:139-46.
3. Prochaska JO, Redding CA, Evers KE. The transtheoretical model and stages of change. Health behavior. 1997:97.
4. Michie S, West R, Campbell R, Brown J, Gainforth H. ABC of behaviour change theories: Silverback publishing; 2014.
5. LaMorte WW. The health belief model. Boston University School of Public Health. 2019.
6. Andersen R, Newman JF. Societal and individual determinants of medical care utilization in the United States. The Milbank Memorial Fund Quarterly Health and Society. 1973:95-124.

**Supplementary Material 2: The Pharmacy Practice Journals, indexed in PubMed, systematically search in conducting this review**

| **Clinical pharmacy practice journals** |
| --- |
| American Journal of Health-System Pharmacy: AJHP |
| Canadian Pharmacists Journal: CPJ |
| Currents in pharmacy teaching & learning |
| European Journal of Hospital Pharmacy |
| European Heart Journal — Cardiovascular Pharmacotherapy |
| Exploratory Research in Clinical and Social Pharmacy |
| Expert opinion on pharmacotherapy |
| Hospital Pharmacy |
| Innovations in Pharmacy |
| Integrated Pharmacy Research & Practice |
| International journal of clinical pharmacy |
| Journal of Basic and Clinical Pharmacy |
| Journal of clinical pharmacy and therapeutics |
| Journal of managed care & specialty pharmacy |
| Journal of managed care pharmacy: JMCP |
| Journal of oncology pharmacy practice |
| Journal of pain & palliative care pharmacotherapy |
| Journal of Pharmacy & Bioallied Sciences |
| Journal of pharmacy practice |
| Journal of pharmacy practice and research |
| Journal of Young Pharmacists: JYP |
| Journal of Research in Pharmacy Practice |
| Journal of the American Pharmacists Association |
| Journal of the American College of Clinical Pharmacy |
| Medicine and Pharmacy Reports |
| Pharmacotherapy |
| Pharmacy and Therapeutics |
| Pharmacy Practice |
| Pharmacy: Journal of Pharmacy Education and Practice |
| Research in social & administrative pharmacy |
| The Annals of pharmacotherapy |
| The American journal of pharmacy benefits |
| The American journal of geriatric pharmacotherapy |
| The Canadian Journal of Hospital Pharmacy |
| The consultant pharmacist |
| The senior care pharmacist |
| The International journal of pharmacy practice |

**Supplementary Material 3: Search strategies applied to the electronic databases included in this review.**

| **Database** | **Search Strategy** | **Filters applied** |
| --- | --- | --- |
| **PubMed** | (pharmacy [Title/Abstract]) AND ((theor*[Title/Abstract]) OR (framework [Title/Abstract])) | Clinical study, clinical trial, clinical trial phase I, clinical trial phase II, clinical trial phase III, clinical trial phase IV, comparative study, controlled clinical trial, evaluation study, Meta-analysis, observational study, pragmatic clinical trial, randomized controlled trial, review, systematic review validation study, English |
| **CENTRAL** | Pharmacy AND (framework OR theories) | **None** |
| **Web of Science** | (TS=(pharmacy)) AND (TS=(framework) OR TS=(theor*)) | English + open access |
| **EBSCO (CINAHL Plus, Britsih libarary index, ERIC)** | AB Pharmacy AND AB (framework OR theory) | Available for free + English + Academic journals |

| **Supplementary Material 3: Full details of included studies.** | | | | | | | | | | | | |
| --- | --- | --- | --- | --- | --- | --- | --- | --- | --- | --- | --- | --- |
| Author, date of publication | Country of the study | Aim of study | Duration of study | Outcome measure(s) | Study setting | Population | Study design | Theory/framework/model used | Purpose of the theory/ framework/model used | Authors rational for the use of theory/ framework/model | Key research findings | Conclusion |
| Kennelty et al, 2015 | USA | (1) examine the barriers and facilitators community pharmacists face when reconciling medications for recently discharged patients; (2) identify pharmacists’ preferred content and modes of information transfer regarding updated medication information for recently discharged patients | December 2012-December 2013 | Barriers and facilitators community pharmacists face when reconciling medications, and pharmacists’ preferred content and modes of information transfer regarding updated medication information for discharged patients | Community pharmacies | 10 community pharmacists | Qualitative, semi-structured interviews | TPB | TPB was used as the guiding theory for constructing initial interview questions, the directed content analysis results are organized based on the TPB constructs | TPB posits that an individual’s attitude toward the behavior, subjective norms, and perceived and actual control of a behavior shape their intent to perform a behavior and the execution of the behavior itself | In the context of the TPB, more barriers than facilitators of reconciling medications were revealed. Themes were categorized as organizational and individual-level themes. Major organizational-level factors affecting the medication reconciliation process included: pharmacy resources, discharge communication, and hospital resources. Major individual-level factors affecting the medication reconciliation process included: pharmacists’ perceived responsibility, relationships, patient perception of pharmacist, and patient characteristics. Interviewed pharmacists consistently responded that several pieces of information items would be helpful when reconciling medications for recently discharged patients, including the hospital medication discharge list and stop orders for discontinued medications | The TPB was useful for identifying barriers and facilitators of medication reconciliation for recently discharged patients from community pharmacists’ perspectives. The elucidation of these specific facilitators and barriers suggest promising avenues for future research interventions to improve exchange of medication information between the community pharmacy, hospitals, and patients |
| Amin et al, 2015 | Egypt | To explore the utility of TPB model in predicting community pharmacists’ Medication Regimen Adjustment (MRA) behavior for patients during Ramadan | November-December 2012 | Community pharmacists’ MRA behavior for patients during Ramadan | Community pharmacies | 363 community pharmacists. 92.9% of the approached pharmacists participated | Quantitative, cross-sectional survey | TPB | Integrated into research question, study design, data collection, analysis & interpretation. This study employed constructs derived from TPB and adapted to the research question following pretesting | TPB implies that individuals carefully consider the available information before acting. According to TPB, attitudes, subjective norms and perceived behavioral control determine the individual’s behavioral intention and consequently determine the likelihood of the individual carrying out that specific behavior | While 94.2% reported performing one or more kinds of MRA around Ramadan for at least one patient, the majority of these were for a small percentage of patients. The most common MRA was changing the frequency of taking the medication followed by the dose of the medication, the dosage form of the medication and the medication itself. Statistically significant predictors of MRA in the final model included patient social pressure (PSP), pharmacist perceived behavioral capability (PBC), pharmacist perceived patient benefit (PPB), initiating communication and the number of working hours | TPB appears to have utility in predicting pharmacists’ behavior. Pharmacists may be open to a larger MRA role than they are currently performing. There is a need to prepare pharmacists to make sure they provide a safe transition for fasting patients into and out of Ramadan |
| DeMik et al, 2013 | USA | To determine whether a correlation exists between existing clinical pharmacy services within a practice-based research network (PBRN) and provider attitudes and beliefs regarding implementing a new pharmacy intervention based on TPB | Not stated | Determinants of behavior theorized to be associated with the implementation of the intervention based on TPB | 32 primary care offices throughout the USA | Physicians returned 321 (35.9%) surveys, while pharmacists returned 40 (75.5%) | Quantitative, cross-sectional survey | TPB | The survey was developed using an operation manual for validated instruments for the TPB obtained from experts in the field. TPB was integrated into the analysis and discussion | TPB has been used to explain physician intentions to perform an activity such as implementation of guidelines. TPB has been used primarily to evaluate implementation of guidelines for chronic conditions; however it has not been used to evaluate prospective clinical pharmacy interventions. Quality improvement strategies for management of chronic diseases (e.g. hypertension, asthma) may benefit from implementation research driven by theory | The Cronbach’s alpha coefficients generally ranged from 0.65 to 0.98. TPB subscale scores were lower in offices rated with lower pharmacy service scores, but these differences were not statistically significant. There was no correlation between clinical pharmacy service score and providers’ TPB subscale scores. In both the hypertension and asthma groups, pharmacists scores were significantly higher than physicians’ scores on the attitudes subscale in the multivariate analysis | Pharmacists consistently scored higher than physicians on the TPB, indicating that they felt the hypertension or asthma intervention would be more straightforward for them to implement than did physicians. There was no significant correlation between clinical pharmacy service scores and attitudes toward implementing a future physician/pharmacist collaborative intervention using the TPB |
| Salgado et al, 2012 | Australia | To explore renal-specialized hospital pharmacists’ intentions to implement pharmacy services in outpatient dialysis centers | October-December 2010 | Measures pharmacists’ views on their potential involvement and perceived ease or difficulty in implementing pharmacy services in outpatient dialysis centers | Society of Hospital Pharmacists of Australia Renal Special Interest Group | 13 Australian renal-specialized hospital pharmacists | Qualitative, semi-structured interviews | TPB | The interview guide was developed based on the TBP. The analysis was also guided by TBP, the coding process began with the identification of discourse passages which fitted each component of the theory, yielding to the main themes of the analysis | TBP has been applied to healthcare research to predict health care professional and patients’ behavioral change | Pharmacists demonstrated positive attitudes towards the implementation of the services. Outcomes expected included benefits to patients, the renal team, and the pharmacy profession, as well as economic savings due to dose optimization and improvement of patients’ adherence. Subjective norm was favorable meaning that nephrologists, nurses and patients were expected to be receptive towards future pharmacy services. Barriers comprised: funding, hospital administrators’ approval, time and staff shortage, academic training, relationship with physicians, and attitudes of pharmacists, renal team, and patients. Facilitators included: having an interview room with access to information sources, consent from the team, access to patients’ profiles, and a full-time pharmacist with a clearly defined role | Pharmacists showed positive attitudes, favorable subjective norm and strong perceived behavioral control, which originated a clear behavioral intention to develop pharmacy services in outpatient dialysis centers |
| Puspitasari et al, 2016 | Australia | (1) pharmacists’ attitudes to delivering cardiovascular disease (CVD) support; (2) environmental factors, influence the provision of CVD care in the community pharmacy setting | Cross-sectional, data from 2014 | The primary dependent variable was the provision of CVD support. There were two secondary dependent variables: the frequency of working with GPs and the level of pharmacies’ involvement in providing enhanced services | Community pharmacies | N = 1350 community pharmacists; response rate 15.8% | Quantitative; cross-sectional survey | TPB | Used in development of data collection tool and then data analysis. A theoretical model was developed from the TPB with additional variables addressing environmental factors, and used as a framework for a survey instrument | The use of a theoretical framework in pharmacy practice studies serves to enrich the value and interpretability of research findings | The model for CVD support provision by demonstrated good fit. Factors found to predict CVD support included: two attitudinal latent factors (“subjective norms of pharmacists’ role in CVD support” and “pharmacists’ perceived responsibilities in CVD support”) and environmental factors i.e. pharmacy infrastructure (documentation and a private area), workload, location; government funded pharmacy practice programs; and pharmacists’ involvement with Continuing Professional Development and attendance at CVD courses | Pharmacists’ attitudes appeared to be the strongest predictor of CVD support provision. TPB was useful in identifying “subjective norms” and “pharmacists’ beliefs” as key constructs of community pharmacists’ attitudes. Community pharmacies would be able to provide such an advanced clinical service if they strongly believed that this was an acknowledged part of their scope of practice, had adequate infrastructure and employed sufficient numbers of pharmacists with appropriate and relevant knowledge |
| Amin et al, 2016 | Egypt | To predict Egyptian community pharmacists’ counseling on oral contraceptives (OCs) while utilizing TPB | Not stated | (1) importance of taking OCs at the same time every day. (2) the appropriate day to start taking OCs. (3) how to act if one or more pills are missed. (4) OCs side effects. The TPB constructs were used as the independent variables | Community pharmacies | Of the 181 pharmacists invited to complete the survey, response rate 93% | Quantitative; cross-sectional survey | TPB | Largely for questionnaire development (extensive, detailed mapping of TPB constructs to questionnaire items) which then followed into the analysis and discussion (although less on the actual theory).  Used constructs derived from TPB and adapted to the research question following pretesting | Provide extensive, detailed text in the introduction on the relevant of TPB to pharmacist counselling in general. Has a figure mapping the TPB constructs with the behavior | Pharmacists indicated they talked to a slightly higher proportion of women about the importance of taking OCs at the same time daily than about topics such as which day to start taking OCs, side effects and what to do when a dose of OCs was missed. Pharmacists’ reported counseling on OCs was positively associated with their perception that women welcomed pharmacist-initiated OC counseling, perceived adequacy of time available to counsel women on OCs and the perceived number of women who asked for their help in selecting an OC without providing a pre- scription in the past week. Pharmacists reported that women’s welcoming pharmacists initiating OC counseling was associated with the pharmacists’ reported percent who asked pharmacists for OC advice out of the last 5 women seeking OC. Male pharmacists were less likely than female pharmacists to report that women welcomed pharmacist-initiated OC counseling | The TPB appears to help predict pharmacists’ OC counseling. There is a need to prepare pharmacists who are frequently requested to assist women with the selection of an oral contraceptive. Interventions that would facilitate women’s requests for information may be valuable to increase pharmacists’ counseling on OCs |
| Lee et al, 2016 | USA | To examine the behavioral influences, motivation, and self-efficacy that may guide a patient’s decision to pick up asthma controller medications from the pharmacy for the treatment of persistent asthma | April- August 2014 | Behavioral beliefs, attitude toward the behavior, normative beliefs and subjective norms | Academic family medicine practice | Patients with asthma treated at one academic family medicine practice.  N=240 patients; 27 individuals consented and completed a survey | Quantitative; cross-sectional survey | TPB | The survey was based upon TPB. It also served as a tool to explore behaviors and attitudes toward asthma in order to begin to understand how they impact the decision to pick up, or acquire a prescribed controller medication | TBP includes key variables that serve as the foundation for an individual’s intention, ultimately resulting in a behavior.  As adherence to medications is dependent upon picking up prescribed medications from the pharmacy, factors that affect filling prescriptions are of particular importance. Utilizing TPB in the context of asthma management, the psychosocial factors that may influence whether an individual picks up a prescription for asthma were explored in this study | Eighteen individuals (67%) were prescribed a controller inhaler in the past year, fourteen of whom picked up their prescription from the pharmacy. Individuals who did not pick up their prescription reported more strongly than those who did that using their inhaler is important. No other statistically significant differences were identified. Regarding control beliefs and perceived behavioral control, participants’ belief that they do not have asthma, not knowing how to use their inhaler(s), use of illicit substance(s) and/or alcohol, and transportation to the pharmacy were not identified as barriers to picking up prescriptions from the pharmacy | Use of an inhaler is important to the patient based upon survey results; however, this belief did not correlate with adherence. Future studies that investigate patient-specific motivators would allow practitioners to better target clinical interventions to improve medication adherence in patients with asthma |
| Amin et al, 2017 | Egypt | To examine factors associated with the unwarranted dispensing of subtherapeutic doses of antibiotics in community pharmacies as part of a cold group or upon direct request from patients among community pharmacy staff | April -December 2016 | Community pharmacy staff’s views on factors associated with the unwarranted dispensing of subtherapeutic doses of antibiotics | Community pharmacies | N= 15, Nine pharmacists and six pharmacy assistants | Qualitative; semi-structures interviews | TPB | TPB constructs framed the different items of the interview question guide. Directed content analysis was performed based on the coding frame. The coding process began with the identification of passages from the transcribed interviews which fitted the theory constructs, yielding to the main themes of the analysis | TPB implies that individuals carefully consider the available information before acting. According to TPB, attitudes, subjective norms and perceived behavioral control determine the behavioral intention and consequently determine the likelihood of the individual carrying out that specific behavior. This construct has been shown to add predictive power to models predicting pharmacists’ intentions to carry out behaviors. The TPB has been useful for qualitative research aiming to explore pharmacists’ behavior | Factors contributing to dispensing antibiotics injudiciously included incorrect beliefs about potential benefit of antibiotics, profit, client pressure, ease of obtaining antibiotics from other pharmacies, inadequate enforcement of the law, pharmacist absenteeism, and assuming that the ‘non malfeasance’ principle is not violated. Reasons for lying to clients about the actual content of CGs included protecting the patient from harm resulting from antibiotic resistance and avoiding a possible argument | Examining attitude, subjective norm, perceived behavioral control and perceived moral obligation provided insight into community pharmacy staff’s behavior related to dispensing subtherapeutic doses of antibiotics injudiciously. Multi-tiered interventions are urgently needed to tackle different factors contributing to this dangerous practice |
| Tan et al, 2016 | Malaysia | To explore the key determinants and mediators of successful implementation of new public pharmaceutical services by investigating the cognitive perspectives of patients’ intentions to adopt with the TPB as the theoretical framework | Not stated | Not stated | Not stated | Patients  No further details provided | Mixed methods; semi-structured interviews, survey | TPB | For the qualitative phase, TPB model serves as the framework for thematic analysis.  Emerging themes from the interview were used to generate a series of hypotheses which was tested using the questionnaire | Intentions to perform behaviors can be predicted from attitudes, subjective norms, and perceived behavioral control. We posit that patient knowledge and expectations about pharmacy value added services (PVAS) may also be significant predictors of intention and may also indirectly influence TPB constructs in affecting intentions | Subjective norms, perceived behavioral control, knowledge and expectations are found to be significant predictors of intentions to adopt PVAS. Knowledge and expectations are found to exert significant indirect effects on intentions | Patient knowledge could be enhanced through appropriate channels and expectations of service quality could be met to increase intentions |
| Nichols et al, 2021 | USA | To characterize community pharmacist preceptors’ experience, clinical and legislative knowledge, attitudes, and behaviors regarding cannabidiol (CBD). The secondary study objective was to identify which of these factors influenced intent to recommend CBD products | January-April 2020 | Not stated | Community pharmacies | Pharmacists, 18 years or older, with active license, practicing in a community pharmacy, and precepted for an accredited school of pharmacy.  N = 2242; responses rate 13.2% | Quantitative; cross-sectional survey | TPB | Survey development | Not stated | For experience items, most respondents (70.7%) reported receiving previous education on CBD. Almost half (48.4%) reported CBD sales in their pharmacies, whereas 89.1% reported answering clinical questions about CBD. For knowledge items, respondents performed poorly on CBD adverse effect and drug interaction items. Many respondents were not comfortable counseling on (49.0%) or recommending (56.1%) CBD products for patient use. Most (74.5%) believed more research was needed before they would feel comfortable recommending CBD products. Most (57.8%) reported not having reliable CBD resources available in their pharmacies. Subjective norms and previous CBD education or personal research were the only factors found to have direct influences on respondents’ intent to recommend CBD products | Opportunities exist to fill knowledge gaps, enhance confidence, and provide desired educational resources for community pharmacist preceptors on CBD products |
| Fleming et al, 2019 | USA | To elicit modal salient beliefs of community pharmacists regarding their willingness to engage patients with suspected controlled substance misuse | Not stated | Beliefs of community pharmacists regarding their willingness to engage patients | Community pharmacies | Community pharmacists | Qualitative; focus groups | TPB | Questionnaire tool developed based on TPB | TPB allows for the assumption that behavioral beliefs, normative beliefs and control beliefs are predictors of that same individual's perceived attitude, subjective norm, and control toward any behavior | The most prevalent behavioral belief was the disadvantage associated with patient confrontations. Pharmacists also believed that engaging patients may cause loss of customers/business but may help patients receive appropriate counseling | Addressing key barriers such as perceptions around loss of customers/business, regulatory barriers and resources- to patient engagement is critical to increasing pharmacists’ willingness to engage patients with potential OUD. |
| Gülpinar et al, 2021 | Turkey | To provide an in-depth understanding of the factors related to the beliefs of community pharmacists on conscientious objection to provide pharmacy services contrary to their personal beliefs based on the TPB | January-May 2019 | Not stated | Community pharmacies | N=25 community pharmacist in Turkey | Qualitative; semi-structures interviews | TPB | Development of interview guide; analysis of interview themes (generation of codes and sub-themes from the same central meaning units) | According to TPB, attitudes, subjective norms, and perceived behavioral control are predictors of individual’s behavioral intention and consequently determine the likelihood of the individual carrying out that specific behavior | Factors affecting pharmacists’ decision to provide pharmacy services when their personal beliefs included moral integrity, consequences on healthcare, profit, patient pressure, precedence of professional values, and care for religious sources. Most pharmacists were against conscientiously objecting to provide services because of possible negative consequences on healthcare. Pharmacists who were willing to act based on their personal beliefs were expecting from various third parties to fulfill certain responsibilities to facilitate to adopt the behavior | This novel study highlights the urgent need for more research and training for community pharmacists serving patients in different socioeconomic contexts in both developed and developing countries |
| Falope et al, 2021 | USA | To explore the perceptions and knowledge of Florida pharmacists in administering inactivated influenza vaccines (IIV) to pregnant women | February - August 2019 | Not stated | Community pharmacies | Licensed pharmacists in the state of Florida | Qualitative; interviews | TPB | Interview guide design | TPB is an intrapersonal level theory with three constructs (behavioral attitudes, social norms, and perceived behavioral control), which all determine why an individual may or may not engage in behavior. For this study, the theory was modified to include knowledge as a construct | The majority of pharmacists (94%) were knowledgeable about the IIV in pregnant women. Participants expressed mixed attitudes, identified barriers and facilitators, and subjective norms influencing vaccine administration in pregnant women. Participants expressed the importance of trust and how that influenced vaccine uptake. Participants also expressed their position not to only provide immunization services but also to counsel and educate patients | There is a need to strengthen immunization services, provided by pharmacists to more individuals, including high-risk groups such as pregnant women |
| Humphries et al, 2018 | Canada | To identify women’s attitudinal, normative, and control beliefs regarding adjuvant endocrine therapy (AET) adherence that could be targeted by an intervention offered in the community pharmacy setting | November 2013 -February 2014 | To elicit general experience with AET and personal beliefs (attitudinal, normative, and control beliefs) regarding AET | Hospital and community pharmacies | Phase 1 (focus groups): N=34, women diagnosed with breast cancer and had their first AET prescription within the last two years.  Phase 2 (interviews): N=9, women with self-report difficulties adhering to AET | Qualitative; focus groups and individual interviews | TPB | To develop a topic guide for the focus groups and interviews | TPB has been widely used to identify the main psychosocial factors influencing health-related behaviors in quantitative and qualitative studies. It is considered as one of the most effective psychosocial theories to predict a behavior, such as medication adherence | Most women had a positive attitude towards AET regardless of their medication-taking behavior. The principal perceived advantage was protection against a recurrence while the principal inconvenience was side effects. Almost everyone approved of the woman taking her medication. The women mentioned facilitating factors to encourage medication-taking behaviors and cope with side effects. For adherent women, having trouble establishing a routine was their main barrier to taking medication. For non-adherent women, it was side effects affecting their quality of life | The use of TPB contributed to the understanding of beliefs that should be targeted in a community pharmacy-based intervention to enhance AET adherence. The identification of priority targets is especially important when designing brief interventions. Focus groups allowed for a general portrait of the experience of women with AET while interviews offered a deeper understanding of non-adherence |
| Adeoye et al, 2018 | USA | To model the association between pharmacy technicians' attitudes and planned behaviors toward participating in MTM and store level MTM completion rates. Secondary objectives included 1) to compare pharmacy technician and pharmacist attitudes and planned behaviors toward participating in MTM and 2) to identify respondent and pharmacy demographic factors associated with MTM completion rates. | February -May 2017 | Not stated | Community pharmacies | N=77 pharmacy technicians (66.4%) and 99 pharmacists (86.8%)  Pharmacy technicians and pharmacists who represented 116 supermarket pharmacy chain locations in a Midwestern division | Quantitative; cross-sectional survey | TPB | Survey development | Not stated | Pharmacy technicians held significantly more positive perceptions about MTM delivery, particularly regarding adequate time and support. However, pharmacy technicians reported having the necessary knowledge and skills significantly less frequently compared with pharmacists. TPB variables for pharmacy technicians were not significant predictors of MTM completion rates. However, pharmacist attitudes, pharmacy technician education, and number of technician hours worked per week were positively associated with MTM completion rates | Pharmacists’ attitudes, pharmacy technician level of education, and number of technician hours worked per week were associated with MTM completion rates |
| Hasan et al, 2019 | USA | To use the TPB to understand factors influencing South Asian consumers’ intention to seek pharmacist-provided medication therapy management services | Not stated | Effects of attitude, subjective norm (SN), perceived behavioral control (PBC), and socio-demographics on South Asian consumers’ intention to seek medication therapy management services | Community pharmacies | Participants who were ≥18 years of age, of South Asian origin, with a previous visit to a pharmacy in the US for a health-related reason, and with ability to read and  comprehend English were recruited from independent pharmacies in New York City | Quantitative; survey | TPB | Items from a previous TPB study on pharmacist intention to provide MTMS were modified to develop items assessing subjective norm in this study. Items to assess the  perceived behavioral control were developed using past literature on TPB questionnaire construction by Ajzen | According to TPB, intention is influenced by attitude towards performing a behavior, subjective norm associated with the behavior, and perceived control over the behavior | Attitude and perceived behavioral controls were significant predictors of intention. None of the socio-demographics were significant predictors of intention | Strategies to make South Asians seek MTMS should focus on creating positive attitudes and removing barriers in seeking MTMS. |
| George et al, 2018 | USA | To explore factors associated with community pharmacists’ beliefs to improve Star Ratings scores using TPB | For 1 year (on 2016) | Beliefs associated with improving Star Ratings | Community pharmacies | N = 4 focus groups with 26 participants  Pharmacists licensed in Oklahoma with a primary practice in the community setting were the target population from which we sampled | Qualitative; focus group | TPB | TPB-based discussion guide, coding of data from the TPB down to specific examples (deductive approach). The use of specific coded nodes to build up to general themes and subthemes (inductive approach) | TPB has been useful in assessing beliefs and predicting health professionals’ behavioral intention. TPB also includes perceived behavioral control, because it was developed as an extension of the Theory of Reasoned Action to account for the influence of volitional control | Four focus groups were conducted with 26 participants in 2 categories: pharmacists with and without experience improving Star Ratings. Pharmacists shared and contrasted in salient, normative, and control beliefs about patient outcomes, data, financial implications, staff, technology, and other stakeholders associated with performance of improving Star Ratings. Themes regarding medication adherence, patient safety, and intention were also found | The TPB was used to explore beliefs of community pharmacists about improving Star Ratings scores. Themes that were identified will assist in future research for measuring intention to improve Star Ratings scores and the development of training and education programs |
| Wash et al, 2022 | Portland | To identify factors that influence pharmacists’ intention to prescribe | May-June, 2019 | Factors that influence pharmacists’ intention to prescribe | Different practice settings | N = 13 participants (12 pharmacists and one fourth-year pharmacy student); response rate 32% | Qualitative; focus groups | TPB | TPB was used to guide this study. Largely in focus groups guide development and data analysis | When following the TPB framework, interviews or focus groups are employed to identify the salient behavioral, normative, and control beliefs underlying the TPB constructs within the target population | The mean score on the awareness assessment was 6.3 ± 0.9 (7 = highest awareness). The majority (76.9%) had previous prescribing experience. Attitudes were found to be shaped by behavioral beliefs related to the impact of pharmacist prescribing on patient-centered care and practice transformation. Normative beliefs were primarily driven by outside groups past experiences. Control beliefs included the following categories: operational readiness; the relationships between comfort, competence, and confidence; and Board of Pharmacy requirements | TPB was useful for exploring pharmacists’ beliefs related to their intention to prescribe using the Formulary and Protocol Compendia (FPC). Determining the relative importance of these factors in a broader population will enable stakeholders to develop interventions to improve uptake of prescribing via the FPC |
| Alenezi et al, 2022 | UK | To explore pharmacists' roles, barriers and determinants related to their involvement in optimizing prescribed opioids for patients with chronic pain | January- May, 2020 | Pharmacists’ perceived roles, barriers and behavioral determinants in relation to opioid therapy optimization | Community pharmacies | N= 20 community pharmacists. The inclusion criterion was currently working in community practice | Qualitative; semi-structures interviews | TDF | TDF informed the development of the interview guide. Excerpts from the interview transcripts were coded into one or more of the TDF domains. Data were coded systematically using a deductive approach, whereby each TDF domain served as a coding category | TDF was developed from a synthesis of psychological theories to help apply theoretical approaches to behavior change interventions. TDF domains represent environmental, cognitive, and social factors that may affect behavior. TDF has been widely used to understand behaviors and implementation challenges in various settings and content areas in health care, including community pharmacies [and hence is suitable in the context of this research | Pharmacists demonstrated desire to contribute to opioid therapy optimization. However, they described that they were often challenged by the lack of relevant knowledge, skills and training, inadequate time and resources, systemic constraints (such as lack of access to medical records and information about diagnosis), and other barriers including relationships with doctors and patients | The contribution of community pharmacists to optimize opioid therapy in CNMP is unclear and impeded by lack of appropriate training and systemic constraints. There is a need to develop innovative practice models by addressing the barriers identified in this study to enhance the contribution of community pharmacists in optimization of opioid therapy for chronic pain |
| Hussein et al, 2021 | Canada | To select the optimal implementation strategies that can address identified factors and accelerate practice change | Not stated | (1) barrier and facilitators influencing the adoption of full scope services among pharmacy professionals; (2) optimal BCTs to address the identified barriers and facilitators | Not specified | All registered users of Pharmacy5in5 (pharmacists and pharmacy technicians)  N=2696; response rate 8.4%, 24 in the interview | Mixed methods; survey, semi-structured interviews | TDF | Questionnaire and interview design and as coding framework for the qualitative analysis; triangulation of the study findings | TDF has a greater capacity to build a behavior change intervention that is more comprehensive and has a wider range of potential BCTs to address barriers and facilitators. Furthermore, mapping BCTs to corresponding TDF domains help maximize the benefit of a theory-based intervention by selecting optimal BCTs that can target pharmacy professionals’ behavior | A number of key barriers were identified on an individual level (e.g., lack of clear professional identity and limited decision-making skills in ambiguous cases) and on an organizational level (e.g., lack of social support from managers and concerns about making more errors with the current workflow). Mapping the barriers and facilitators to BCTs yielded 18 BCTs to support the adoption of full scope services, including modeling, rehearsal/practice, and social support | This study highlighted several barriers that need to be addressed to facilitate pharmacy professionals working to their full scope, including professional collaboration, professional identity, and adequate training. A comprehensive intervention combining skills training with modeling, social support, and decision-making tools could encourage practice change |
| Lindner et al, 2022 | Australia | To investigate Austrian community pharmacists’ willingness to administer immunizations in the future | Not stated | Percentage of employed Austrian community pharmacists willing to administer immunizations, a ranking of relevant requirements and barriers to implementation of an immunization service and desired training specifications | Community pharmacies | N=3086; response rate 12.3%  All registered employed community pharmacists in Austria | Quantitative; cross-sectional survey | TDF | The questionnaire was based on best practice guidelines and the TDF.  The following domains of the TDF were addressed in the study: skills, professional role and identity (section 1); beliefs about capabilities, intentions goals, environmental context and resources, social influences (section 2); goals (section 3). | TDF is an integrative framework validated as a method ‘for theoretically assessing implementation problems’ | Willingness to administer immunizations after appropriate training and legislative regulation was stated by 82.6% (n=314) of participants. It was demonstrated that pharmacists willing to immunize were significantly younger than their counterpart (38 [IQR 31–49] years vs. 45 [IQR 37.5–54] years; OR 1.06; 1.03–1.09, 95% CI; p<0.001). ‘Legal liability’ was considered the most critical barrier to service implementation, ‘seeing blood’ and ‘close patient contact’ as least critical. Pharmacists not willing to immunize showed a higher probability to evaluate personnel resources (OR 2.98; 1.35–6.58, 95% CI; p=0.007), close patient contact (OR 2.79; 1.46–5.34, 95% CI; p=0.002) and management of side effects (OR 2.62; 1.21–5.67, 95% CI; p=0.015) as (highly) critical. The majority assessed the ‘right timing for training’ to be after the foundation training with a 2-yearly renewal | Austrian community pharmacists show a strong willingness to administer immunizations while highlighting important requirements and barriers towards service implementation |
| Rushworth et al, 2018 | UK | To quantify issues of access to GPs, community pharmacies and prescribed medicines in older people resident in the Scottish Highlands. | Not stated | Not stated | General public | N =1042, response rate  54.2% | Quantitative; survey | TDF | The attitudinal statements were developed with reference to the TDF | TDF was derived from 33 psychological theories and 128 theoretical constructs, which are organized into 14 overarching domains. The statements were developed around those domains most relevant to access to prescribed medicines (e.g. memory, attention and decision processes social influences, emotions etc.) | The majority reported convenient access to GPs (89.1%) and community pharmacies (84.3%). Older age respondents were more likely to state that their access to GP services was not convenient and those in rural areas to community pharmacies. For access to prescribed medicines, those in poorer health and taking five or more regular prescribed medicines were more likely to state access not convenient. PCA identified three components of beliefs of capabilities, emotions and memory. Those with poorer health had more negative scores for all. Those reporting issues of access to prescribed medicines had more negative scores for beliefs of capabilities while those of older age, living alone, and taking five or more regular prescribed medicines had more negative scores for emotions | While the majority of respondents have convenient access to their GP practice, pharmacy and prescribed medicines, there is a need for further review of the pharmaceutical care of those of older age with poorer health, living alone in the more remote and rural areas and taking five or more prescribed medicines |
| Mohammed et al, 2021 | Saudi Arabia | To explore the experiences, perceptions and barriers of Saudi pharmacists about their uptake of non-traditional roles using TDF | Not stated | pharmacist’s perceptions, current opportunities and key challenges towards the uptake of non-traditional roles | Not specified | N=14  Qualified Saudi pharmacists who were in the UK as part of their further study. | Qualitative; interviews | TDF | Identified themes were triangulated with the domains in TDF | TDF enables the identification of appropriate components of planned behavioral interventions, the barriers and enablers which need to be addressed, and the way behavior changes brought through the interventions can be measured and understood | Participants showed an overall positive attitude towards the uptake of non-traditional roles. Participants felt that there was wider support available for pharmacists at the policy level to uptake non-traditional roles. However, a need for greater recognition of roles by other healthcare professionals and patients were identified. Participants alluded to reluctance of some physicians to take on board the suggestions from a pharmacist. Key barriers to uptake of non-traditional roles were related to environmental context and resources domain of TDF. For example, participants discussed the need for even further practical experiences during their undergraduate degree to become ready to adopt non-traditional roles in clinical practice | Participants of this theoretically informed qualitative study showed an overall positive attitude towards the way pharmacy practice is progressing in Saudi Arabia and their uptake of non-traditional roles. However, there is a need to improve interdisciplinary working, patient awareness of pharmacist competencies and their educational preparedness in furthering their uptake of non-traditional roles. Addressing such barriers and promoting uptake of novel roles by pharmacists is imperative in the context of emerging COVID-19 and future pandemics |
| Seubert et al, 2018 | Not stated (likely Australia) | To describe the development of a behavior change intervention to enhance information exchange between pharmacy personnel and consumers during OTC consultations in community pharmacies | Not stated | Development of a behavior change intervention | Community pharmacies | Community pharmacist, pharmacy staff and consumers | Mixed methods; systematic review, focus groups, intervention development, intervention feasibility study | TDF | Coding of focus groups findings was based on TDF domains.  A behavioral diagnosis on the target behavior, as described in the BCW | BCW is a validated framework developed to assist researchers to apply the COM-B model in any setting to develop an intervention strategy. TDF is a validated derivation of the COM-B that provides a more detailed understanding of determinants of behavior from which an intervention strategy can be developed | Education, persuasion, environmental restructuring, and modelling were determined to be potential intervention functions | A systematic, theoretically underpinned approach was applied to develop candidate interventions to promote information exchange in OTC consultations |
| Seubert et al, 2019 | Australia | To explore the feasibility of interventions using situational cues to promote information exchange between pharmacy personnel and consumers, during OTC consultations | Not stated | Phase 1 (literature review): enhance communication during OTC consultations.  Phase 2 (focus groups): barriers and facilitators for information exchange during OTC consultations  Phase 3 (intervention strategy development): intervention functions and the resulting BCTs that would most suitably address these barriers were identified  Phase 4 (intervention feasibility study): ffeasibility study to determine if the intervention strategy could work | Community pharmacies | Pharmacy staff and consumers | Mixed methods study; audio-recording, OTC consultations, consumer questionnaires and interviews, and pharmacy personnel interviews | TDF | Phase 2: The results of the focus group discussions were coded and mapped to the TDF  Phase 3: using the BCW framework, intervention functions and the resulting BCTs that would most suitably address these barriers were identified | BCW assists health researchers to apply the COM–B model of behavior in any setting, to develop an intervention strategy. The TDF allows for a more detailed understanding of the determinants of behavior. The BCW links intervention functions, which are the active components that can be used in an intervention strategy and can be observed and replicated, with BCTs | Pharmacy personnel perceived that the badges positively impacted consumers’ ability to identify the position of personnel they engaged with. Data collection methods  were deemed practical and acceptable | Effective interventions are necessary to promote information exchange to enhance appropriate management in community pharmacies |
| Isenor et al, 2018 | Canada | To identify the relationship between barriers and facilitators to pharmacist prescribing and self-reported prescribing activity using the TDF(v2) | Not stated | 1) the extent of self-reported pharmacist prescribing, 2) pharmacists' perceptions on their prescribing role, 3) barriers and facilitators to pharmacist prescribing using the TDF (v2), 4) relationship between respondents' perceptions of factors that may influence pharmacist prescribing by TDF domain and self-reported prescribing activity. | All licensed pharmacists that are members of the Pharmacy Association of Nova Scotia (PANS) | N=87 (11.5%)  All licensed pharmacists that are members of the PANS | Quantitative; cross-sectional survey | TDF (version 2) | To map each survey question according to the TDF domains | Understanding environment and context is critical for implementing new practices and using the TDF can identify barriers and facilitators at the individual pharmacist and pharmacy team levels, as well as in the broader context including ethical, legal, political and financial dimensions, but also those that are less quantifiable, such as nonfinancial organizational incentives, and peer pressure | The three domains that respondents most positively associated with prescribing were Knowledge (84 %), Reinforcement (81 %) and Intentions (78 %). The largest effect on prescribing activity was the Skills domain (OR 4.41, 95% CI, 1.34-14.47) | Applying the TDF(v2), our study identified barriers and facilitators at the individual practitioner (e.g. knowledge and skills), pharmacy (e.g. adequate time and staffing for prescribing), regulatory (e.g. concerns related to liability) and health care system (e.g. physician relationship, limited reimbursement) levels.  The TDF(v2) domains provided a valuable structure for comparing respondent beliefs and attitudes about a primary outcome variable (prescribing activity). This allowed for the simple logistic regressions to be built, but the inherent co-linearity between TDF(v2) domains prevents a multivariate model from being produced, which makes it challenging to study how the TDF(v2) domains may be related to one another. |
| Patton et al, 2021 | Ireland | (1) Explore barriers/facilitators influencing community pharmacists’ provision of medication adherence support (MAS) to older patients prescribed multiple medications; (2) Identify theoretical domains to target for behavior change; (3) Select BCTs to deliver to pharmacists to enhance MAS provision | Not stated | (1) Explore determinants that influence community pharmacists' provision of MAS to older patients; (2) Identify exactly what could be targeted to change pharmacists' behavior; (3) Select BCT to deliver as part of a training package and implementation strategies to include in future studies | Community pharmacies | Phase 1 (interviews): N=15, pharmacists working in a registered community pharmacy in Northern Ireland. Phase 2 (survey): N=143 (27.4%), community pharmacists working in the region of Northern Ireland | Mixed methods; semi-structured interviews, cross-sectional survey | TDF (version 1) | Phase 1 (interviews): interview topic guide Phase 2 (survey): development of the questionnaire  Data analysis: TDF domains served as coding categories Triangulation of findings: a domain was deemed important if it was frequently coded in the qualitative analysis and/or the findings from the quantitative analysis  Mapping of findings: TDF guided selection of BCT | Can act as a ‘theoretical lens’ to explore health providers’ clinical behaviors and gain a more comprehensive understanding of the key influences of that behavior. The selection of TDF1 was supported by a discriminant content validity exercise undertaken by Huijq et al. which recommended ‘keeping to the 12 original domains as a basis for the development of TDF questionnaires’ | Phase 1 (interviews): Barriers and facilitators included inadequate remuneration, time and knowledge of solutions and professional confidence. Phase 2 (survey): Potential barriers included inadequate training in motivational techniques and difficulties with decision-making Triangulation: seven domains (e.g. skills, motivation/goals) were identified as targets and mapped across to 18 BCTs (e.g. behavioral practice/rehearsal, prompts/cues). | The use of a comprehensive theoretical model of behavior change has helped go beyond identifying the barriers commonly cited in the literature (e.g. time, reimbursement). This theory-based approach has facilitated an exploration of other important areas to target for behavior change such as pharmacists' skills and approaches to decision-making |
| Cardwell et al, 2018 | Northern Ireland | To explore community pharmacists’ views on the facilitators and barriers towards the utilization of a screening tool as a guide to conducting structured medicines use review (MURs) | Not stated | Not stated | Community pharmacies | N = 18  Community pharmacists working in pharmacies affiliated with the Community Pharmacy Placement Network (which provides a structured placement program for pharmacy students), who had undertaken the necessary training in the provision of MURs | Qualitative; semi-structures interviews | TDF | Semi-structured interviews were conducted with community pharmacists using a TDF-based topic guide | Not stated | Based on the analysis of 15 interviews, 11 TDF domains (‘Knowledge’, ‘Skills’, ‘Social and professional role and identity’, ‘Beliefs about capabilities’, ‘Beliefs about consequences’, ‘Reinforcement’, ‘Goals’, ‘Memory, attention and decision process’, ‘Environmental context and resources’, ‘Social influences’, ‘Behavioral regulation’) were deemed relevant. Facilitators included: knowledge of patients, clinical knowledge, perceived professional role, patients’ clinical outcomes, influence of peers. Barriers included: prioritization of other clinical activities, inability to access patients’ clinical information, perceived alienation from the primary healthcare team and staffing issues | Using the TDF, key facilitators and barriers were identified in the use of a screening tool as a guide to conducting MURs. These findings may assist in further development of MURs as a means to optimize patients’ medicines use |
| Paudyal et al, 2019 | UK | To determine community pharmacists’ training, experiences and behavioral determinants in counselling and management of homeless population | November 2016 -March 2017 | Pharmacists’ perspectives, pharmacists’ training, pharmacists’ experiences and behavioral determinants | Community pharmacies | Community pharmacists | Quantitative; survey | TDF (version 2) | To construct questionnaire items | Not stated | Less than a third (n=101, 32.2%) indicated that they knew where to refer a homeless patient for social support. Broaching the subject of homelessness was outside their comfort zone (n=139, 44.3%). Only four (1.2%) respondents could correctly answer all knowledge assessment questions | Community pharmacist identified lack of education, training opportunities and guidelines in counselling and management of homeless persons requiring interventions to improve skills and opportunities |
| Gemmeke et al, 2021 | Holland | To assess community pharmacists’ perceptions on providing fall prevention services, and to identify their barriers and facilitators in offering these fall prevention services including deprescribing of fall risk-increasing drugs (FRIDs) | February - June 2020 | Not stated | Community pharmacies | N= 466 pharmacists, response rate 44%. 16 pharmacists were interviewed | Mixed methods; survey, interviews | COM-B model | Applied to analyze and interpret the qualitative data | The COM-B model is a widely used behavioral change theory and therefore a suitable framework to identify needs to change. The COM-B model has been used to describe healthcare providers’ dependencies to express a desired behavior | Pharmacists are motivated to provide fall prevention services, but their capability differs. They have had diverse opportunities to provide fall prevention, with key facilitators being efficient collaboration and establishment of multidisciplinary agreements. Pharmacists indicated that major barriers were patient’s unwillingness to cease medication, the complexity of deprescribing, limited goal-setting behavior, a lack of time, and a lack of financial compensation. It has previously been reported that pharmacists believe they should be involved in fall prevention; however, only a minority have actually been involved | Community pharmacists deem themselves capable of providing fall prevention services, and they are motivated to do so, particularly by deprescribing FRIDs. However, they perceive the decision-making of FRID deprescribing as complex due to difficulties in weighing fall risk against treatment benefit. Pharmacists believe they could provide better fall prevention services in collaboration with other disciplines |
| Hattingh et al, 2019 | Australia | To explore the factors that contributed to the successful implementation and ongoing provision of enhanced and extended services in Western Australian community pharmacies | October 2017 -February 2018 | The factors that contributed to the successful implementation and ongoing provision of enhanced and extended services | Community pharmacies | N=28 pharmacists. Pharmacists working in WA who were providing enhanced and/or extended professional services | Qualitative; semi-structures interviews | COM-B model | The COM-B model was applied post hoc to the thematic analysis to explore whether there was an overlap between themes and the model | COM-B model provides a framework for understanding behavior and incorporates the practice context ‘opportunity component’ and more internal aspects ‘motivation and capability components’. This model highlights the need to consider both pharmacist capabilities and motivation as well as local opportunities and the policy framework in planning to introduce new services | Factors that impacted on provision of services: 1) pharmacist characteristics, 2) local needs, structures and support, and 3) an enabling practice framework | Both pharmacy and pharmacist aspects should be considered during implementation and maintenance of new professional services |
| Bertilsson et al, 2021 | Australia | To identify factors affecting recruitment of patients in community pharmacies participating in a multicenter trial of a pharmacy asthma service in Australia (Pharmacy Trial Program – Asthma and Rhinitis Control (PTP-ARC) | Not stated | 1. Capability: physical or psychological  2. Opportunity: physical or social 3. Motivation: reflective or automated | Community pharmacies | N=47 out of 50 eligible pharmacists  All pharmacists involved in the PTP-ARC trial whose staff had not successfully recruited any patients or whose pharmacy had no active participants in the trial | Qualitative; semi-structures interviews | COM-B model | To analyze findings. The exploration of pharmacists’ experiences applied COM-B model | The COM-B model was developed to analyze behavior change when implementing new interventions. The COM-B model has been widely used in implementation research, including in the general implementation of asthma guidelines in community pharmacy and clinical pathways in complex tertiary clinical settings | Seventeen factors were isolated and mapped to COM-B model. Psychological capability (recruitment hesitancy, research literacy and health literacy), physical capability (technological barriers, staffing issues and pharmacy busyness), physical opportunity (patient busyness, trial timing, study protocol, support and location), social opportunity (health literacy and supportive milieu), reflective motivation (incentive for participation, simplification) and automatic motivation (patient attitudes and pharmacist-felt experience) were factors affecting pharmacists’ participation. Challenges included: issues with the software, unfamiliarity with research procedures generally (and specifically with the PTP-ARC protocols), the patients’ lack of interest and pharmacists’ lack of time | Incorporating the COM-B model in the BCW suggests interventions and policies likely to be effective when addressing the highlighted barriers. To propel evidence-based trials towards practice implementation, user-friendly software, pharmacists’ training on research and patient-engagement and adequate remuneration to address pharmacist time issues need to be key foci for health services design and implementation research |
| Pinto et al, 2006 | USA | (1) design and test a reliable and valid survey instrument for assessing patients’ perceptions of diabetes-related pharmaceutical care services. (2) determine factors affecting patient retention in pharmaceutical care services | February-April 2001 | (1) Patients’ perceptions of diabetes-related pharmaceutical care services. (2) Factors affecting patient retention in pharmaceutical care services | Community pharmacies | Type 1 and type 2 diabetes patients who had received diabetes-related pharmaceutical care within community pharmacy. Total of 71 surveys returned (46.67%) | Quantitative, cross-sectional survey | HBM | The survey was developed based on the constructs of the HBM. Based on the factor analysis results, the items that formed the constructs of the HBM were summed to get a total score value for each factor | Leaders in the field of psychosocial research state that as a starting point, at a minimum, a combination of constructs from the HBM needs to be tested for understanding health behaviors | In the absence of pharmaceutical care, patients felt susceptible to at least one of 8 diabetes-related conditions. For these conditions, about 48% to 95% of patients perceived that their threat had reduced because of pharmaceutical care. Accordingly, more than half perceived the services as beneficial, with counseling for blood sugar monitoring rated as the most beneficial. All respondents rated the services as helpful, and 64 intended to continue regular utilization. Overall helpfulness of the service and patient retention were positively correlated. Perceived susceptibility predicted perceived threat reduction. Perceived threat reduction, blood sugar monitoring, and overall helpfulness of the service predicted patient retention in the service. Factor analysis extracted 4 factors: perceived threat reduction, perceived susceptibility, perceived benefits, and blood sugar monitoring | Two key constructs of the HBM influence use of diabetes related pharmaceutical care services: perceived susceptibility and threat reduction. In an effort to increase patient retention, pharmacists need to assess patient perceptions and structure their services to address patient perceptions and concerns |
| Alatawi et al, 2016 | Saudi Arabia | To test the HBM as a predictor of type 2 diabetes (T2D) medication adherence as measured with multiple new or established self-report adherence instruments. The specific objectives: (1) assess self-report of medication-taking in a Saudi T2D convenience sample, (2) investigate self-reported HBM constructs for T2D, its complications, and medication-taking in this sample, and (3) test the ability for self-reported health beliefs to predict specific medication-taking behaviors among the sample | June 1-July 24, 2014 | Three measures collected self-report of medication adherence: new multi-dimensional adherence measure (MDAM), previously validated stage of change, and medication-taking recall- 7days (MTR-7) | Outpatient pharmacy | N= 222; response rate 87.4%  Patients with T2D, over the age of 18, and who were on at least one prescribed diabetes medication | Quantitative; cross-sectional survey | HBM | Largely in questionnaire design, data interpretation and discussion. The authors state: responses to the HBM items comprise the HBM construct summary scores for Perceived Susceptibility, Perceived Severity, Perceived Benefits, Self-efficacy, and Cues to Action; these were adapted from portions of the 18-item Expanded Health Belief Model Questionnaire (EHBMQ) for T2D. | HBM provides an established theoretical framework for studying adherence and suggests that adherence with a health behavior is explained by associations among six health beliefs/values constructs | For adherence, most reported taking the prescribed dose every time taken; however, 60% were not taking it the prescribed number of times per day and 50%, not the prescribed time of day (interval). Over 40% reported low adherence on stage of change and MTR-7. Perceived susceptibility, perceived medication benefits, and self-efficacy were significant HBM predictors for medication adherence | The MDAM has research and practice potential because it evaluates sub-behaviors of medication-taking separately and as a score. Patient perceptions and beliefs should be assessed as part of a patient-centered medication adherence intervention. |
| Ziaei et al, 2015 | UK | To investigate Internationally Trained Pharmacists (ITPs’) perceptions of their communication proficiency and the resultant impact on patient safety | May-July 2010 | ITPs’ perceptions of their communication proficiency and the resultant impact on patient safety | Community & hospital pharmacies | 31 European Economic Area (EEA) and 11 non-EEA pharmacists practicing in the UK | Qualitative, focus groups | Model of Communicative Proficiency (MCP) | MCP was used as a simple instrument to understand the findings. Using the model in this way allowed researchers to offer suggestions for change, or how, or where to concentrate efforts for improvement and/or training | Based on MCP and further research, the Canadian English Language Benchmark Assessment for Nurses (CELBAN) was successfully designed, which was the first nationally validated, occupation-specific language assessment tool designed to assess the language proficiency of internationally trained nurses seeking licensure in Canada | ITPs experienced communication difficulties through new dialects, use of idioms and colloquial language in their workplace. The differences between the “BBC English” they learned formally and the “Street English” used in GB also led to difficulties. Culture was also recognized as an important aspect of communication. ITPs in this study were adamant that communication difficulty did not compromise patient safety | Communicative deficiency of ITPs arose primarily from two sources: linguistic competence and socio-cultural competence. These deficiencies could have negative implications for patient safety. The findings of this study should be taken into account when designing adaptation programs for ITPs |
| Odukoya et al, 2014 | USA | To describe the process used by community pharmacy staff to detect, explain, and correct e-prescription errors. | October 2012-April 2013 | The process used by community pharmacy staff to detect, explain, and correct e-prescription errors | Community pharmacies | N = 13 pharmacists and 14 technicians | Mixed methods; observations, interviews, focus groups | Three-step error recovery model | The error recovery model constructs were used to develop the interview guide and helped to create probes to the interview questions. The codes were conceptualized by using constructs of the three-step error recovery framework, that is, detection, explanation, and correction | The concept of error recovery was employed to guide the exploration of the e-prescription error recovery process in community pharmacies. The application of human factors concepts offers a novel approach to improving patient safety in community pharmacies. To explore e-prescription error recovery in community pharmacies, a human factors approach was employed. Most error recovery studies have been carried out in other industries, with minimal error recovery research in health care settings | Most of the e-prescription errors were detected during the entering of information into the pharmacy system. These errors were detected by both pharmacists and technicians using a variety of strategies which included: (1) performing double checks of e-prescription information; (2) printing the e-prescription to paper and confirming the information on the computer screen with information from the paper printout; and (3) using colored pens to highlight important information. Strategies used for explaining errors included: (1) careful review of patient’s medication history; (2) pharmacist consultation with patients; (3) consultation with another pharmacy team member; and (4) use of online resources. In order to correct e-prescription errors, participants made educated guesses of the prescriber’s intent or contacted the prescriber via telephone or fax. When e-prescription errors were encountered in the community pharmacies, the primary goal of participants was to get the order right for patients by verifying the prescriber’s intent | Pharmacists and technicians play an important role in preventing e-prescription errors through the detection of errors and the verification of prescribers’ intent |
| Desai et al, 2015 | USA | (1) explore the characteristics of internet buyers of medicines/vitamins. (2) examine the association between health care use and buying medicines /vitamins online drawing on Andersen’s health care utilization framework. (3) examine the factors predicting discussion of internet information with health providers | Cross-sectional, data from 2007 | (1) characteristics of internet buyers of medicines/vitamins. (2) Association between health care use and buying medicines/vitamins online. (3) Factors predicting discussion of internet information with health providers | Used data from National Cancer Institute’s Health Information National Trends Survey | N = 7674 buyers  Model 1 used data from 4428 buyers and Model 2 from 5030 buyers | Quantitative; cross-sectional survey | Andersen Behavior Model | Guided independent variable selection to identify factors associated with accessing medications/vitamins online for Model 1. Additionally a parallel model (Model 2), was developed to identify factors associated with discussions of online information. This model emerged from findings from Model 1 and included most independent variables from Model 1 with the inclusion of perceived quality of communication | Using a model like the Andersen Model could be extremely important for exploratory research in this area as little is known about online behavior. Having the Andersen Model as the guiding framework would help strengthen the study and provide structure. Moreover this framework allows evaluating health behaviors in the model which included use of health services which were recognized as an important aspect to health. Additionally, the framework acknowledges the influence of other behaviors on actions. Hence this framework allowed for evaluating one behavior (buying online) in the context of other behaviors (use of health services and talking to providers) | About 85% of online buyers had a regular provider, but only 39% talked to the provider about online information even though most (93.7%) visited the provider R1 times/year. Multivariate analyses found internet health product consumers were more likely to be over 50 years old, have insurance and discuss the internet with their provider than non-internet health product consumers. Moreover, discussion of internet information was more likely if consumers had a regular provider and perceived their communication to be at least fair or good in general | There is a clear association of online buying with age, frequency of visits and discussing online information Although most online buyers visited a provider in the prior year, only a minority discussed the internet with them. This suggests a missed opportunity for providers to help patients navigate internet buying, particularly if they are a patient’s regular provider and the patient perceives their communication as good |
| Waddell et al, 2017 | Australia | To map the leadership and management domain of the Australian Advanced Pharmacy Practice Framework (APPF) against the model of transformational leadership and make comment on the potential utility of the APPF to develop advanced practitioners in the area of leadership and management | Not stated | Extent of mapping of the framework to the elements of the model | Mapping in Australia | Not applicable | Mapping | Alimo-Metcalfe and Alban-Metcalfe model of transformational leadership | Used as a tool to map the Australian framework | In the introduction, the authors describe several different leadership models and the development of these over time. The model used was selected on the basis that it was developed and validated within the UK public service, particularly the National Health Service (NHS) and included middle-level managers.  The authors stated that this model of leadership was determined by Health Workforce Australia (HWA) to be the most suitable to meeting the needs of leaders in the Australian healthcare system | There were broad differences between the APPF and a model of transformational leadership. The APPF focuses heavily on ‘organizational’ skills such as strategic vision and setting direction, while the model of transformational leadership focused on the ‘people skills’ of effective leaders. The APPF mapped primarily to the ‘Leading the Organization’ dimensions of transformational leadership | There are significant differences between the leadership and management competencies of the APPF and the dimensions of transformational leadership. Future work on the competency standards for general and advanced levels of practice should include or refer to an evidence-based model of transformational leadership |
| Qudah et al, 2021 | Not applicable | To identify barriers and facilitators of patients' engagement in pharmacy consultations and investigate the impact of patients on pharmacists' counseling behavior | Not applicable | (1) predictors of patient participation in medication counselling, (2) the influence of patients on pharmacist counseling behavior | Community pharmacies | Patients participating in patient-pharmacist communication | Systematic review | Street’s Linguistic Model of Patient Participation in Care (LM) | Data synthesis (mapping) | Street’s LM framework suggests that the term participation in the pharmaceutical consultation means more than simply seeking advice. Understanding factors and patient characteristics affecting pharmacist-patient consults has the potential to help develop better training and education programs and resources for both pharmacists and patients to ensure patient-centered care and consultations | Fifty studies from 1983 to 2019, including 37 using self-reported data, were identified. Patient involvement in patient-pharmacist communication was influenced by enabling factors for participation ranged from patients' beliefs and past experiences to demographic characteristics such as gender and age. Pharmacists' participative behavior with patients was positively associated with patients' engagement and perceived patient cues in the conversation | Pharmacy encounters should no longer be viewed as controlled simply by pharmacists’ expertise and agendas. Patient factors  also appear to influence patient-pharmacist communication. Additional research needs to address the identified facilitators and barriers to enhance patient participation and pharmacist counseling behavior |
| Chevalier B et al, 2017 | Australia | To explore hospital pharmacists’ and patients’ views about what constitutes effective communication exchanges between pharmacists and patients | November 2015 - April 2016 | (1) What aspects of a conversation make it effective; (2) What’s important to you; (3) What makes a good conversation; (4) Why do you think it was effective; (5) What would have made it more effective | Inpatient wards and outpatient clinics | N= 12 pharmacists and 48 patients  Pharmacists who provided clinical pharmacy services. Eligible patients were prescribed three or more medications to manage a chronic disease(s) and had been admitted to a clinical area in which a study pharmacist practiced | Qualitative; semi-structures interviews | Communication Accommodation Theory (CAT) | Pharmacists’ and patients’ opinions of effective pharmacist–patient exchanges were analyzed using a process of inductive thematic analysis and then mapped to the CAT strategies | CAT has been widely used in healthcare communication research. Five CAT strategies will facilitate the analysis of pharmacists’ and patients’ perspectives of effective conversations | An overall shared goal was the assurance of patients’ confidence in managing their medications at home. To facilitate this, patients focused mainly on pharmacists’ delivery of medication information and interpersonal behaviors. Pharmacists’ themes included building rapport, but also emphasized patients’ understanding of their medications and their level of engagement as indicators of patients’ confidence in self-managing their therapy. Participants provided rich exemplars for each of the themes | Pharmacists and patients provided valuable insights about what makes pharmacist–patient interactions effective. Patient-identified preferences for pharmacist–patient exchanges may help guide pharmacy students and practitioners to engage patients in effective conversations |
| Murad et al, 2017 | Canada | To determine face needs, threats and the strategic communication strategies used to address these within community pharmacist-patient interactions | Not stated | (1) main activities of community pharmacists (2) interactional contexts appear to contain face implications (3) types of face needs and threats implicated in these contexts | Community pharmacies | 25 audio recordings of patient-pharmacist interactions in eight different pharmacies were used in this study | Exploratory descriptive design | Face-work theory | As a guiding framework and for data analysis | Face-work involves a set of coordinated communication practices in which communicators build, maintain, protect, or threaten personal dignity, honor, and respect. In Face-work theory, the concept of face represents claimed social image in the interaction | Looking at the process of 25 patient-pharmacist interactions as they occurred has provided insight into face concerns within the main activities of pharmacist practice in community pharmacies. Pharmacists engaged in assessing patient knowledge, providing medication information, and investigating patients' medication use and self-management activities. Pharmacists followed common work patterns when communicating with their patients. The interactional contexts with face implications reflect how the patient and the pharmacist approached each other to accomplish their goals and their reaction to the flow of events in the encounter | Face-work Theory is a novel approach to understand processes and out- comes of patient-pharmacist interactions in community pharmacies. Linking speech acts with face needs and threats may help to elucidate how pharmacist-patient interactions achieve both task oriented and interpersonal goals |
| Luder et al, 2016 | USA | To survey new enrollees in a community pharmacy, employer-based diabetes and hypertension  coaching program to describe the characteristics, health beliefs, and cues to action of newly enrolled participants | November 2011-November 2012 | The survey analyzed 12 constructs of the HBM, TPB, and Theory of Reasoned Action (TRA) | Community pharmacies | N= 154 patients  All newly enrolled patients in the diabetes and hypertension coaching programs. | Quantitative; cross-sectional survey | HBM, TPB, and TRA | Largely for questionnaire development | HBM, TPB, and TRA are effective models for explaining behavior and predicting patient preventative actions. No studies were found which describe the patient behaviors and health beliefs that predict enrollment into disease management programs in a community pharmacy | The strongest cue to action for enrollment was the financial incentives offered by the employer. White patients were significantly more motivated by financial incentives | A top factor motivating patients to enroll in a disease management coaching program was the receipt of financial incentives. Significant differences in HBM, TPB, and TRA responses were seen for patients with different demographics |
| Luke et al, 2021 | Canada | To elucidate the factors influencing the integration of pharmacogenomics (PGx) testing by pharmacists in their practices and to use the BCW approach to inform future intervention options to support pharmacists with this integration | February-April 2017 | Not stated | Pharmacists who completed the PRIME training program irrespective of setting | 10 out of the 21 pharmacists who completed the PRIME training program participated in the interviews | Qualitative; semi-structures interviews | TDF and COM-B model | TDF domains were applied as deductive codes to the interview data to describe the subjective accounts of the participants’ experiences with PGx testing in practice. Themes relating to the barriers and facilitators to implementing PGx services were then mapped onto the corresponding components of the COM-B model | TDF can guide the identification of the determinants of service implementation that exist at multiple levels (e.g., the individual level, the organizational level, and the health-system level), however, the majority of its domains relate to factors that govern behavior often conventionally considered to occur at an individual level. A unique feature of the TDF is that it complements COM-B model for understanding behavior. The domains of the TDF have been mapped onto the components of the COM-B model, each of which represents antecedents for behavior and is, thus, a potential target for interventions aimed at promoting behavior change | The application of the TDF framework revealed “internal” facilitators and barriers with respect to integrating PGx testing in pharmacy practice. Successful implementation was dependent on features of the pharmacists’ professional identities, practice environments, self-confidence, and the perceived benefits of adding PGx testing to their practice. Potential interventions to improve the implementation of the PGx service included preparing pharmacists for managing an increased patient load, helping pharmacists navigate the software and technology requirements associated with the PGx service, and streamlining workflows and documentation requirements | Pharmacists must possess a combination of factors for implementation to be successful. The TDF model was used to articulate the barriers and facilitators of service implementation. Enablement, environmental restructuring, and persuasion are the potential intervention types suggested by the BCW to address these barriers and equip primary care pharmacists with the capability, opportunity, and motivation to implement and deliver a PGx service in their practice |
| Viegas et al, 2021 | Portugal | To characterize physical activity promotion actions taking place in the Portuguese community pharmacies, as well as the major facilitators and barriers faced by pharmacists in their daily practice | November - December 2018 | Not stated | Community pharmacies | N = 2745 community pharmacists; response rate 14.4% | Quantitative; cross-sectional survey | COM-B model and TDF | Questionnaire development | Theories of behavior change can support the identification of the main drivers and challenges faced by pharmacists | Three out of four participants reported to promote physical activity in their daily routine, of which 87.7% reported doing it in only a few attendances. The majority (92.3%) mentioned to provide information orally, with walking being the activity most promoted (99.4%). More active and younger pharmacists were more likely to promote physical activity. Nearly all pharmacists (98.7%) believed it was important or very important to practice regular physical activity for the health, but only 41.4% of the respondents were able to correctly identify the WHO general recommendations for physical activity. The lack of coordination with other healthcare professionals, lack of interest by customers and lack of time were the main barriers, all scoring above the scale mid-point | Physical activity promotion in the Portuguese community pharmacies is still not present as daily activity. Younger pharmacists seem to better understand this need and could easily integrate this practice in their daily routine. Possibilities for including pharmacies and pharmacists as promoters of physical activity in the primary health care sector in the future are discussed in the light of these findings |
| Faisal et al, 2021 | Canada | To explore factors affecting implementation of a real-time adherence-monitoring, multidose-dispensing system in community pharmacies | November 2019 to June 2020 | Not stated | Not stated | Pharmacists and pharmacy assistants who packaged and dispensed medications in smart multidose packages and monitored real-time medication intake via a web-portal remotely for older adults | Mixed methods; semi-structured interview, survey | The Technology Acceptance Model (TAM), TPB, and COM-B model | interview development; analysis (mapping) of themes | It has been argued that TAM alone cannot predict healthcare providers’ beliefs about the use of health-related technology, therefore, we used an integrated approach of combing two behavior theories (TPB and COM-B model) with TAM framework to add rigor to the study | The study results indicated that pharmacists valued the availability of real-time medication intake data and perceived that it could be a useful tool to aid in clinical decision-making related to therapy. The study identified numerous factors that were not solely related to pharmacy workflow; they included pharmacists’ perceived patient determinants and product features which may impact the implementation of such interventions in community pharmacy settings | Products with real-time adherence monitoring capabilities are valued by pharmacists. A careful assessment of infrastructure—including pharmacy workload, manpower and financial resources—is imperative for successful implementation of such interventions in a community pharmacy setting |
| Abdu-Aguye et al, 2021 | Nigeria | To understand barriers/facilitators to optimal medication counselling by conducting a behavioral analysis using the COM-B model, TDF, and BCW as a basis for identifying evidence-based intervention strategies and policy categories that could be used to improve outpatient medication counselling by pharmacy staff in hospital settings | January-March 2020 | Thirteen questions under four main themes, and explored areas including dispenser views on their current medication counselling practices and their perceptions of selected facilitators and barriers to effective patient counselling | Outpatient pharmacies located within hospitals | N=25 Pharmacists or pharmacy technicians who have worked for at least 6 months, and are actively involved in day-to-day outpatient medication counselling | Qualitative; semi-structures interviews | COM-B model and TDF | TDF & COM-B: Interview guide design.  Data analysis: Themes identified from interviews were deductively coded using TDF domains as a priori codes, and then mapped on to the relevant COM-B component. The behavioral analysis was conducted using the BCW to identify areas requiring change to improve counselling. Once these areas were identified, evidence-based intervention and policy functions required to support these changes were then selected from the BCW | The TDF may be used together with the COM-B model, and both of these frameworks can help researchers better understand the determinants of different behaviors, and clearly identify targets for behavioral change interventions. The outer parts of the wheel then provide a systematic way of identifying relevant intervention and policy categories based on what is understood about the behavior(s) underlying them | Findings revealed shortfalls in pharmacy staff capability, opportunity and motivation with respect to outpatient medication counselling. To improve their counselling behaviors, change was identified as necessary in eight TDF domains namely ‘knowledge’, ‘interpersonal skills’, ‘memory’ ‘environmental context’, ‘social influences’, ‘intentions’, ‘reinforcement’ and ‘beliefs about capabilities’. Seven intervention functions including ‘education’, ‘training’, ‘modelling’, ‘enablement’ and ‘environmental restructuring’, in addition to three policy categories (‘guidelines’, ‘regulations’ and ‘environmental/social planning’) were also identified as relevant to future intervention design. | The use of BCW/TDF combined framework likely led to the identification of a wider range of potential barriers to medication counselling than has been reported in the literature.  Various factors were identified as affecting medication counselling by the pharmacy staff, with several of them requiring changes if counselling was to be improved upon. Multi-component interventions combining several of these intervention functions are recommended for hospital authorities and other relevant stakeholders to improve outpatient medication counselling |
| Bright et al, 2021 | USA | To identify patient perceptions related to pharmacogenomic testing in the community pharmacy setting | 2-17 June, 2016 | Interview questions include content about performing pharmacogenomic testing and interpreting pharmacogenomic testing for the purpose of clinical decision making | Community pharmacies | N=19 Adult patients taking either a selective serotonin reuptake inhibitor (SSRI) and/or clopidogrel/prasugrel/ticagrelor | Qualitative; semi-structures interviews | Rubin and Rubin framework and TPB | Interview guide: Rubin and Rubin framework  Interview questions: TPB | Not stated | Four themes related to patient perceptions of pharmacogenomic testing were consistently observed across multiple interviews: 1) trust, 2) experience, 3) risk/benefit, and 4) clarity. | These themes may influence the desire to pursue pharmacogenomic testing |
| Jonkman et al, 2020 | Namibia | 1) Identify patient-reported barriers and facilitators to managing chronic non-communicable diseases (NCDs); 2) characterize common patient-reported medication and health related needs with chronic NCDs | September 2018-January 2019 | 1) Barriers and facilitators to managing chronic NCDs; 2) patient-reported medication and health related needs of Namibians with chronic NCDs | Public hospitals, private hospitals, and community pharmacies | N=20 Adults, with at least one chronic medical problem (e.g. hypertension), who were coming to pick up an outpatient prescription | Qualitative; semi-structures interviews | HBM, TPB, and the Explanatory Models of Illness (EMI) | To develop the interview guide. The constructs identified of particular importance were obtained from the HBM and TBM. Concepts from the EMI model were used to elicit cultural perceptions around the chronic disease | Not stated | Themes identified included: 1) participants were motivated to seek care when they were symptomatic; 2) participants felt motivated to care for their condition to improve their own lives and their families for their family’s sake; 3) participants integrated information from a variety of sources into their disease knowledge; 4) participants describe wanting to be more engaged in managing their health and wanting support to help manage their condition; 5) participants describe awareness of lifestyle changes necessary to improve health, but face many barriers to achieving them | This study identified key factors that are essential for pharmacists and other healthcare professionals to be aware of to support patients who are diagnosed with an NCD. Healthcare providers should consider strategies to engage patients to harness their motivations, enhance health education, and create systems to reduce barriers to addressing lifestyle |
| Fingleton et al, 2018 | UK | To establish how non-prescription medicine (NPM) dependence is treated by doctors in specialist substance misuse treatment services and to identify perceived barriers to providing treatment | Survey: May-October 2015; Interview: date not specified | To establish current practice and whether changes to service provision are needed to facilitate treatment | Specialist substance misuse treatment services | Doctors | Mixed methods; survey, semi-structured interviews | TDF and COM-B | A selection of survey questions was derived from COM-B model to determine behaviors needed to change to facilitate the treatment of NPM dependence. The interview topic guide was developed based on TDF | TDF allows to understand behavior-change processes, design interventions to enhance healthcare practice and assess implementation problems. The COM-B model enables understanding of influences on behavior | Most respondents were unaware of specific guidelines for the treatment of non-prescription medicine dependence. The most frequently identified barriers to providing treatment identified by interviewees were limited resources or capacity and the challenges presented by this client group | There is a clear need for specific clinical guidelines for the treatment of NPM dependence; research into effective treatment methods for this client group may be required to ensure that guidelines are evidence-based |
| Okuyan et al, 2022 | Turkey | To determine the intention to receive COVID-19 vaccine and to identify the factors related to it based on the HBM framework among Turkish pharmacists | December 2020 - January 2021 | Intention to receive COVID-19 vaccine and to factors related to it among Turkish pharmacists | Community pharmacies | N = 2631 pharmacists; response rate 36.5% | Quantitative; cross-sectional survey | Transtheoretical Model of Behavior Change and HBM | Questionnaire development | Not stated | Around 74.7% pharmacists had an intention to receive the COVID-19 vaccine. In model 1, perceived susceptibility, perceived severity, perceived benefits, and perceived barriers were associated with their intention to receive the COVID-19 vaccine. In model 2, the intention to receive COVID-19 vaccine was associated with being male, years of experience in the professional field, not having contracted COVID-19, having a pharmacy staff who had contracted COVID-19, and having had received seasonal fu shot within the previous year | This study highlights the factors related to the intention of the pharmacists to receive COVID-19 vaccines. HBM is the strongest predictor for vaccination intention and could be used to develop behavioral change techniques to promote vaccination |
|  | TBP: Theory of Planned Behavior; HBM: Health Belief Model; COM-B: Capability, Opportunity, and Motivation Behavior model; BCW: Behavior Change Wheel; TDF: Theoretical Domain Framework; BCT: Behavior Change Techniques; MTM: medication therapy management; OTC: over the counter; GP: general practitioners. | | | | | | | | | | | |
